# Glutelin subtype-dependent protein localization in rice grain evidenced by immunodetection analyses

**DOI:** 10.1007/s11103-019-00855-5

**Published:** 2019-03-25

**Authors:** Kei Takahashi, Hiromi Kohno, Tomomichi Kanabayashi, Masaki Okuda

**Affiliations:** 1 National Research Institute of Brewing, 3-7-1 Kagamiyama, Higashi-hiroshima, Hiroshima, 739-0046 Japan; 2 Biopathology Institute Co., Ltd, 1200-2, Ohara Kunisakicho, Kunisaki-city, Oita 873-0511 Japan

**Keywords:** Glutelin, Rice grain, Seed storage protein, Protein body type-II (PB-II), Endosperm, *Yamadanishiki*

## Abstract

**Key message:**

GluA and GluB-4/5 glutelin subfamilies are mainly localized to outer region of the endosperm, particularly in its ventral side, in rice grain, but GluC is localized to throughout the endosperm.

**Abstract:**

The major seed storage protein in rice (*Oryza sativa*) is glutelin, which forms a vacuole-derived protein body type-II. Glutelins are encoded by multiple genes, and generally comprise four protein subfamilies, namely, GluA, GluB, GluC, and GluD: however, the localization pattern of glutelin in rice grains remains obscure. In this study, we investigated the localization pattern of five subtypes of the glutelin protein in rice grains using glutelin-subtype specific antibodies. Immunoblot analysis against sequentially polished rice flour fractions from three crop years and seven japonica rice varieties revealed that GluA was strongly localized in the outer region of the endosperm, including the subaleurone layer, whereas GluC was distributed throughout the endosperm. Among the glutelin subtypes, GluA was mostly found in the outer region of the rice grain, followed by GluB-4/5, GluB-1, GluD, and GluC. Immunofluorescence labeling microscopy analysis using immature rice seeds clearly revealed that the localization pattern of GluC and GluD was completely different from that of GluA and GluB. Expression levels of all glutelins, particularly GluA, GluB-1, and GluB-4/5, were stronger on the ventral than dorsal side in rice grains. These results provide strong and consistent evidence that glutelins localize to the rice grain in a subfamily-dependent manner.

**Electronic supplementary material:**

The online version of this article (10.1007/s11103-019-00855-5) contains supplementary material, which is available to authorized users.

## Introduction

Rice is a staple crop for almost half of the world’s population. Asian rice (*Oryza sativa*) contains two major subspecies: *japonica* and *indica* (Wing et al. 2018). Short grain variety of *japonica* is the primary form cultivated in Japan. Starch makes up 70% of *japonica* brown rice, followed by 15% moisture and 7–9% protein. Rice proteins provide valuable nutrient for humans (Juliano 1993). However, it is well known that rice proteins negatively impact the taste of cooked rice, and excessive rice protein is thought to lower the eating quality of rice in Asian countries, such as Japan, China, and Korea (Song et al. 2012). In addition, lower protein contents are desirable for enhancing the quality of processed foods using rice in some cases.

The rice seed storage proteins include glutelin, prolamin, and α-globulin. Glutelin is the major rice storage protein, which accounts for 50% of the total seed protein content. Rice seed storage proteins are synthesized on the rough endoplasmic reticulum (ER) and subsequently are translocated into the ER lumen. Prolamin is stored in protein body type-I (PB-I), which are ER-derived spherical compartments. Glutelin and α-globulin are transferred to vacuoles via the Golgi apparatus (Fukuda et al. 2011, 2013; Tian et al. 2013) and stored in the irregularly shaped protein body type-II (PB-II) derived from protein storage vacuoles (PSVs) (Tanaka et al. 1980; Yamagata and Tanaka 1986). Glutelin polypeptide is cleaved into an N-terminal half (acidic subunit) and a C-terminal half (basic subunit) in the vacuole by an aspartic protease (Wang et al. 2009; Kumamaru et al. 2010). They are conjugated intra-molecularly and inter-molecularly by disulfide bonds to form a higher structural conformation (Katsube-Tanaka et al. 2004, 2010; Kawagoe et al. 2005; Motoyama et al. 2009), and they accumulate with α-globulin in PSVs. Glutelin is encoded by a multigene family, and the molecular type has been studied by many researchers before the whole rice genome was revealed (Takaiwa et al. 1987, 1991; Masumura et al. 1989; Takaiwa and Oono 1991; Mitsukawa et al. 1998; Qu et al. 2002; Kusaba et al. 2003). Recently, it has been reported the glutelins consist of 12 full-length gene copies, which are classified into 4 subfamilies (GluA, GluB, GluC, and GluD) based on the similarity of their amino acid sequence (Kawakatsu et al. 2008; Kawakatsu and Takaiwa 2010).

On the basis of assessments via a colorimetric promoter activity assay using β-glucuronidase (GUS) reporter, glutelins are believed to localize in the aleuronal and subaleuronal layers and starchy endosperm in developing rice seeds (Wu et al. 1998; Qu and Takaiwa 2004; Qu et al. 2008; Kawakatsu et al. 2008). Interestingly, GUS expression patterns directed by glutelin promoters vary considerably depending on the specific glutelin subfamily (Qu and Takaiwa 2004; Qu et al. 2008; Kawakatsu et al. 2008). For example, *GluA-1, GluA-2*, and *GluA-3* promoters drive reporter activity in the aleurone and subaleurone layers, and to a lesser extent, in the outer region of the endosperm. However, *GluB-5* and *GluC* promoters were equally active in the endosperm at 17 days after flowering (DAF) in immature rice seeds (Qu et al. 2008). In addition, immunofluorescence assays using anti-glutelin antibodies showed that the glutelin protein signal was considerably stronger in the external compared with the internal region of the endosperm (Ohdaira et al. 2011). Despite these advances, the localization pattern of glutelin in rice grains remains unclear, due to the following reasons: (i) low resolution of the colorimetric promoter activity assay, (ii) variation in signal activity due to the promoter length used and the presence or absence of the untranslated nucleotide sequence (Liu et al. 2010), and (iii) the use of non-specific glutelin antibodies.

Glutelins are readily digested by protease enzymes, yielding a large amount of amino acids and peptides. Glutelins are digested in various biological situations/processes, such as digestive organs in the human body, embryo germination in rice, and by microorganisms involved in the rice wine (Japanese sake) brewing process. Japanese sake is a traditional alcoholic beverage in Japan, and the major ingredients used for brewing sake are polished rice (*O. sativa japonica* sp.), rice mold (*Aspergillus oryzae*), and sake yeast (*Saccharomyces cerevisiae*) (Kitagaki and Takagi 2013). According to the 2018 annual governmental report of the national tax agency in Japan, sake brewing required 241 kilotonnes of brown rice per year in Japan, and the average rice-polishing ratio (the ratio [w/w] of polished rice to original brown rice) of rice used in sake brewing was 64%. Although brown rice grains used for cooking are usually polished to remove approximately 8–10% of the rice bran (90–92% rice-polishing ratio), the brown rice grains used for sake brewing are polished to remove up to 36% of the outer rice portion, including rice bran. The polishing procedure decreases crude protein, fat, mineral, and vitamin levels, while increasing the proportion of starch in rice (Kanauchi 2013). A lower level of protein in polished rice is thought to be necessary for premium *ginjo* sake brewing. Rice protein, including glutelin and its degradation products, affects the fermentation process and the taste of sake (Takahashi et al. 2012, 2016; Okuda et al. 2016; Takahashi and Kohno 2016). However, the quantity and specific subtypes of glutelin in highly polished rice grains are poorly understood. Therefore, it is necessary to investigate the localization pattern of glutelin subtypes in rice grains.

In this study, we generated five types of anti-glutelin antibodies, including at least one for each glutelin subfamily. Glutelin localization in rice grains was biochemically analyzed using rice powder fractions with these antibodies. In addition, we used immunofluorescence microscopy to reveal glutelin subtype-dependent localization. Biochemical and cellular biological approaches produced concordant results that glutelins localize to the rice grain in a subfamily-dependent manner.

## Materials and methods

### Plant materials

Seven cultivars of brown rice were obtained from the Society for the Studies of Brewer’s Rice and harvested between 2009 and 2011. These included *Yamadanishiki* (Higashi-Hiroshima, Hiroshima), *Nipponbare* (Higashi-Hiroshima, Hiroshima), *Gohyakumangoku* (Kitakata, Fukushima), *Dewasansan* (Sakata, Yamagata), *Koshihikari* (Chiba, Chiba), *Dewanosato* (Higashi-Okitama, Yamagata), and *Yumenokaori* (Kitakata, Fukushima).

### Polyclonal antibodies

**Fig. 1:**
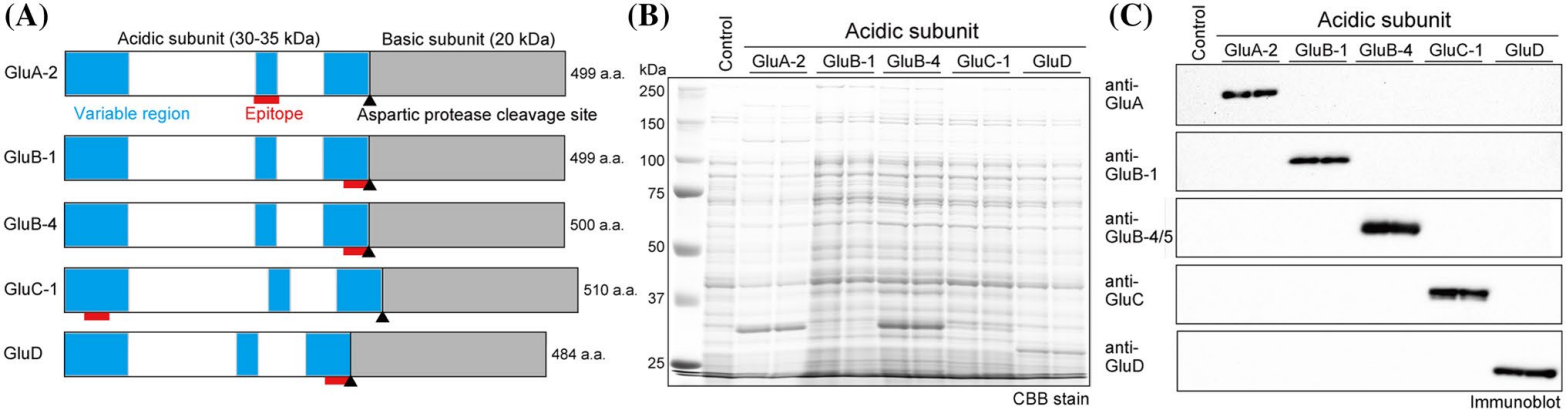
Generation of five glutelin subtype-specific antibodies. **a** Epitope locations in the five glutelin subfamilies. Epitope peptide sequences are depicted by red bars. Variable regions with the glutelin acidic subunit, which have lower homology among glutelin subfamilies, are depicted in blue. Glutelin basic subunits are shown in gray. The aspartic protease cleavage site is depicted as a black triangle. **b** Expression of recombinant glutelin acidic subunit protein in *E. coli* (Rosetta-gami B, DE3). A protein extracted sample from two representative transformants and stained with CBB after SDS–PAGE is shown. **c** Immunoblot analysis of five recombinant glutelin acidic subunits expressed in *E. coli* (Rosetta-gami B, DE3) using each glutelin subtype antibodies. The protein applied and transformants used are the same as (**b**)

Five types of polyclonal anti-glutelin antibodies were generated against the following synthetic peptides: the cysteine + 210–225 amino acid sequence of GluA-2 (C + KRNPQAYRREVEEWSQ) from accession number X05664, the cysteine + 288–301 amino acid sequence of GluB-1 (C + QVQYSERQQTSSRW) from accession number X54314, the 32–48 amino acid sequence of GluC-1 (LQSPRGFRGDQDSRHQC) from accession number AB016501, the cysteine + 267–284 amino acid sequence of GluD (C + RQEEHRQYQQVQYREGQY) from accession number AY429650 (Kawakatsu et al. 2008), and the 286–302 amino acid sequence of GluB-4/5 (AQYQVQYSEEQQPSTRC) from accession number CAA32566. The locations of the synthetic peptides used as epitopes in glutelin molecules are presented in Fig. 1. All antibodies were raised in a rabbit, except for anti-GluB-4/5, which was raised in rats. Immunoglobulins (IgGs) were purified from rabbit and rat serum by affinity chromatography using the corresponding synthetic peptide conjugated columns. The generated antibodies were subdivided and maintained at − 80 °C until required.

Anti-TOC75 (AS06 150) and anti-ADP-glucose pyrophosphorylase (AS11 1739) antibodies produced in rabbits were purchased from Agrisera antibodies (Vännas, Sweden).

### Expression of glutelin acidic subunits in *Escherichia coli*

Five recombinant codon-optimized glutelin acidic subunits were synthesized (GenScript, NJ, USA) and inserted to a pET-11a vector. These included (i) 1–930 nucleotide sequence of GluA-2 from rice annotation project (RAP) locus LOC_Os10g26060, (ii) 1–918 nucleotide sequence of GluB-1 from Os02g0249900, (iii) 1–921 nucleotide sequence of GluB-4 from RAP locus LOC_Os02g16830, (iv) 1–966 nucleotide sequence of GluC-1 from RAP locus LOC_Os02g25640, and (v) the 1–867 nucleotide sequence of GluD from RAP locus LOC_Os02g15090. The generated plasmids were transformed into Rossetta-gami B *Escherichia coli* BL21 cells (DE3; Sigma-Aldrich, MO, USA). Glutelin proteins were induced by the addition of isopropyl-1-thio-β-D-galactoside (IPTG).

### Preparation of fractionated rice powder from endosperms

One hundred grams of brown rice was polished to 90% (w/w) of the original weight using a home rice polisher (Yamamoto Electric Corp., Fukushima, Japan). Ninety grams of the 90% polished rice was sequentially polished to 70, 50, and 30 g with the aid of a grain test mill (Chiyoda Engineering, Inc., Hiroshima, Japan) using an HS-4 whetstone roll#60 and a roll mesh. The rotation speed was 1800 rpm for the first 10 min to eliminate the rice embryo. Thereafter, the rotation speed was reduced to 1400 rpm. Rice powder produced at each step was sampled as 90–70%, 70–50%, 50–30% rice powder. The remaining rice grains (30% rice-polishing ratio) were pulverized using a CMT-100 auto-grinder.

### Rice protein extraction from endosperm

Rice powder (20 mg fresh weight) was suspended in 600 μL of protein extraction buffer containing 4 M urea, 6% SDS, 50 mM Tris-HCl (pH 6.8), 20% glycerol, 2% DTT, 10 μg/mL leupeptin, and 100 μg/mL PMSF. The rice powder suspension was passed through a 23-G syringe 20 times and mixed gently at 100 rpm on a bioshaker for 1 h. After flushing, an 80 μL aliquot of supernatant was mixed with 20 μL of 5 × Ling’s solubilizing buffer containing 200 mM DTT, 50 mM Tris–HCl (pH 8.0), 5 mM EDTA, 10% SDS, and 50% sucrose. Protein was denatured at 50 °C for 15 min. One hundred microliters of 2 × urea buffer (10 M urea, 40 mM DTT, 10 mM Tris-HCl [pH 8.0], 1 mM EDTA, 2% SDS, and 10% sucrose) was then added to the sample.

### SDS-PAGE and immunoblot analysis

SDS–PAGE was performed using 15% polyacrylamide gels (ATTO) with protein size markers (Bio-Rad, Hercules, MA, USA). After separation, the proteins were stained with 0.1% Coomassie brilliant blue (CBB) R-250. The gel was destained for at least 3 h before being directly scanned using an EPSON GT 900 scanner (Nagano, Japan).

For immunoblot analysis, proteins were transferred from gels to 0.22-μm pore PVDF membranes (Whatman) at 12 V, 60 mA, for 30 min. The membrane was soaked in blocking buffer (Nacalai tesque, Inc., Kyoto, Japan). After incubation with target primary antibodies (1/1000–1/8000 dilution), the membrane was washed three times with PBS-containing Tween 20. The membrane was incubated with secondary antibodies conjugated with HRP before being washed a further three times with PBS-containing Tween 20. Proteins were detected by chemical luminescence (ECL prime, GE Healthcare Japan, Tokyo, Japan) using either an LAS 1000 (GE Healthcare) or ChemiDoc™ MP imaging system (Bio-Rad). QuantityOne software ver. 4.6.9. (Bio-Rad) was used to determine the intensity of detected protein bands.

### Crude fractionation and protein extraction of the aleurone layer, the subaleurone layer, and embryo

Brown rice (150 g) was polished to 95% (w/w) of the original weight using a domestic rice polisher set at extremely low speed. The embryo and the aleurone layer containing the pericarp and testa fractions in the rice powder were removed from the subaleurone layer fraction by vibrating separation. The separated embryo and aleurone fractions were sampled. The remaining 95% polished rice was polished further to 90% (w/w) of the original weight using a domestic rice polisher set at low speed. After removing the embryo and pieces of broken rice, the subaleurone layer fraction was collected.

Each fraction (20 mg of fresh weight) was suspended in 600 μL of the protein extraction buffer and broken using a Biomasher II device (Nippi, Tokyo, Japan). The suspension was passed through a 23-G syringe 20 times, and gently mixed at 100 rpm on a bioshaker for 1 h. After centrifugation, an 80 μL aliquot of supernatant was mixed with 20 μL of the 5 × Ling’s solubilizing buffer. Protein was denatured at 50 °C for 15 min. Urea buffer (2×, 100 μL) was then added to the sample.

### Sampling of rice seeds at different developmental stages

In 2012, *Yamadanishiki* and *Nipponbare* cultivars were planted in a paddy field at the National Research Institute of Brewing (NRIB; Higashi-Hiroshima, Hiroshima). With the exception of the apex caryopsis, caryopses from primary rachis branches were sampled in the morning at 6, 8, 10, 12, 17, 22, and 27 DAF and immediately stripped of the hulls before being stored at − 80 °C until required.

### Quantitative real-time PCR

Total RNA of developing rice grains was prepared from 8 to 12 grains. Developing rice grains were crushed in liquid nitrogen, after which total RNA was extracted from the resulting powders using an RNeasy Plant Mini kit (Qiagen). The quality of extracted RNA was assessed using a NanoDrop 1000 Spectrophotometer, Agilent 2100 Bioanalyzer, and Agilent RNA 6000 Nano Kit. cDNA was synthesized using a SuperScript VILO cDNA Synthesis Kit (Invitrogen). Quantitative real-time PCR was performed in a 20 μL volume using a TaqMan kit (Life Technologies) and an ABI PRISM 7900HT PCR system (Life Technologies). The Assay IDs (Life Technologies), including primer sets and reporter sequences for “Custom TaqMan® Gene Expression Assays,” used in quantitative real time-PCR are summarized in Supplementary Table 1. The 17S rRNA gene was used as control. Relative expression quantity for each gene was calculated by the ΔΔCt method using ABI PRISM SDS 2.1.

### Glutelin detection by immunofluorescence microscopy

Developing rice seeds, *Yamadanishiki, Nipponbare*, and *Gohyakumangoku* (sampled in 2014 NRIB) at 17 DAF were examined with immunofluorescence microscopy. For immunofluorescence staining, seed sections (approximately 4 μm thick) embedded in paraffin were deparaffinized with xylene followed by a stepwise change of ethanol. The slides were exposed to 1% hydrogen peroxide/methanol for 30 min to block endogenous peroxidase activity before being washed with water and Tris-buffered saline (TBS). The sections were incubated with anti-glutelin antibodies (1/100 diluted) or an anti-TOC75 antibody (1/100 diluted) overnight. As secondary antibodies, we used Alexa Fluor 594 donkey anti-rat IgG (against GluB-4/5), Alexa Fluor 488 donkey anti-rabbit IgG (against GluA, GluB-1, GluC, GluD, and TOC75), or Alexa Fluor 488 donkey anti-rat IgG (against GluB-4/5) at a 1:50 dilution. The sections were treated with SlowFade Gold Antifade reagent with DAPI (Molecular Probes). Immunofluorescence microscopy analysis was performed using a BX-52 microscope (OLYMPUS, Tokyo, Japan). Images of whole sections of rice grain were captured using a VS120 virtual slide system (OLYMPUS) and analyzed using an OlyVIA software (OLYMPUS). Tissue assignment of the immature rice grain was referred to a previous report (Wu et al. 2016).

## Results

### Establishment of glutelin subtype specific antibody

To reveal the localization pattern of rice glutelin in the rice grain, specific antibodies for each glutelin subtype were created. Recombinant codon-optimized glutelin acidic subunits (Fig. 1a) were expressed in *E. coli* for all but GluB-1 and GluC-1 acidic subunits, which were difficult to express (Fig. 1b) even with an *E. coli* cell line, such as Rossetta-gami B (pLysS) and OverExpress™ C43 (DE3) cells produced by Lucigen (WI, USA). Antibodies against GluA, GluB-1, GluB-4/5, GluC, and GluD specifically recognized each glutelin acidic subunit protein (Fig. 1c).

### Localization pattern of crude protein in rice grains

**Fig. 2:**
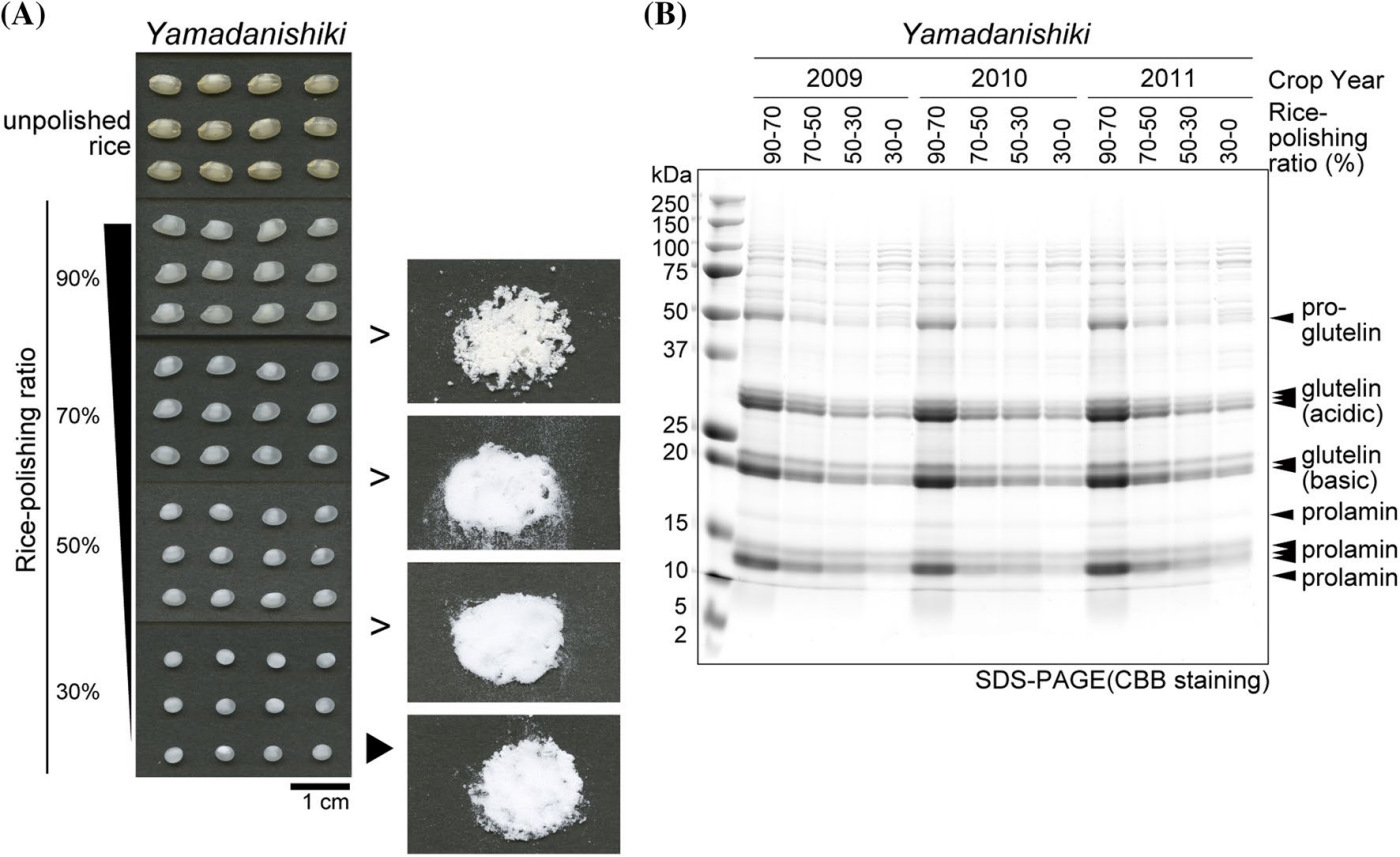
Preparation of fractionated rice grain powder and CBB staining of total rice proteins. **a** Brown rice was polished to 90% (w/w) using a domestic rice polisher. Ninety grams of 90% polished rice was sequentially polished to 70 g, 50 g, and 30 g using a grain test mill. Rice powder produced at each step was sampled as 90–70%, 70–50%, 50–30% rice powder, respectively. The remaining 30 g of rice (30% rice-polishing ratio) was pulverized using an auto-grinder CMT-100. **b** Proteins extracted from fractionated rice powder from three different crop years (2009, 2010, and 2011) were separated by SDS-PAGE and CBB stained. Each lane contains the same weight of rice powder

To analyze rice glutelin localization biochemically, we generated fractionated rice powders. Mature brown rice was polished to a 90% rice-polishing ratio, and then to a 30% rice-polishing ratio. The remaining 30% of the grains were pulverized. Finally, four types of grain powder sample were obtained: 90–70%, 70–50%, 50–30%, and 30–0% fractions (Fig. 2a).

Proteins were extracted from these powders across three different crop years, 2009, 2010, and 2011, and analyzed by SDS-PAGE (Fig. 2b and Supplementary Fig. 1). The 2009 growing season was characterized by relatively low summer temperatures (an average temperature of 21.6 °C during 30 days after the heading of *Yamadanishiki* rice), whereas the 2010 growing season had relatively high temperatures (an average temperature of 26.2 °C during 30 days after the heading of *Yamadanishiki* rice) (Supplementary Table 2). The average temperature in 2011 was intermediate between 2009 and 2010. High temperatures during the grain-filling period have been reported to increase the PB-II/PB-I ratio (Ashida et al. 2013) due to glutelin upregulation and prolamin downregulation (Yamakawa et al. 2007; Lin et al. 2010). Our results confirm that the glutelin/prolamin ratio was higher in rice harvested in 2010 than in rice harvested in 2009 (data not shown, unpublished data), but the localization pattern did not differ (Fig. 2b and Supplementary Fig. 1). As expected, total protein comprising primarily of glutelins and prolamins was more abundant in the external region than in the internal region of these rice grains. However, several specific proteins appeared to be distributed equally in rice grains. Protein patterns on SDS-PAGE were roughly similar across the rice cultivars examined (Supplementary Fig. 1). The *Yamadanishiki* cultivar is the most popular variety for sake brewing, with grains that genetically express the white-core trait at a frequency of 60–70%. We sorted *Yamadanishiki* cultivar grains (white-core grain and non-white core grain), but no obvious difference was detected in crude protein levels between the white and non-white core *Yamadanishiki* rice grains by SDS-PAGE (Supplementary Fig. 1g, h).

### Localization pattern of glutelin in rice grains determined by immunoblot analysis

**Fig. 3:**
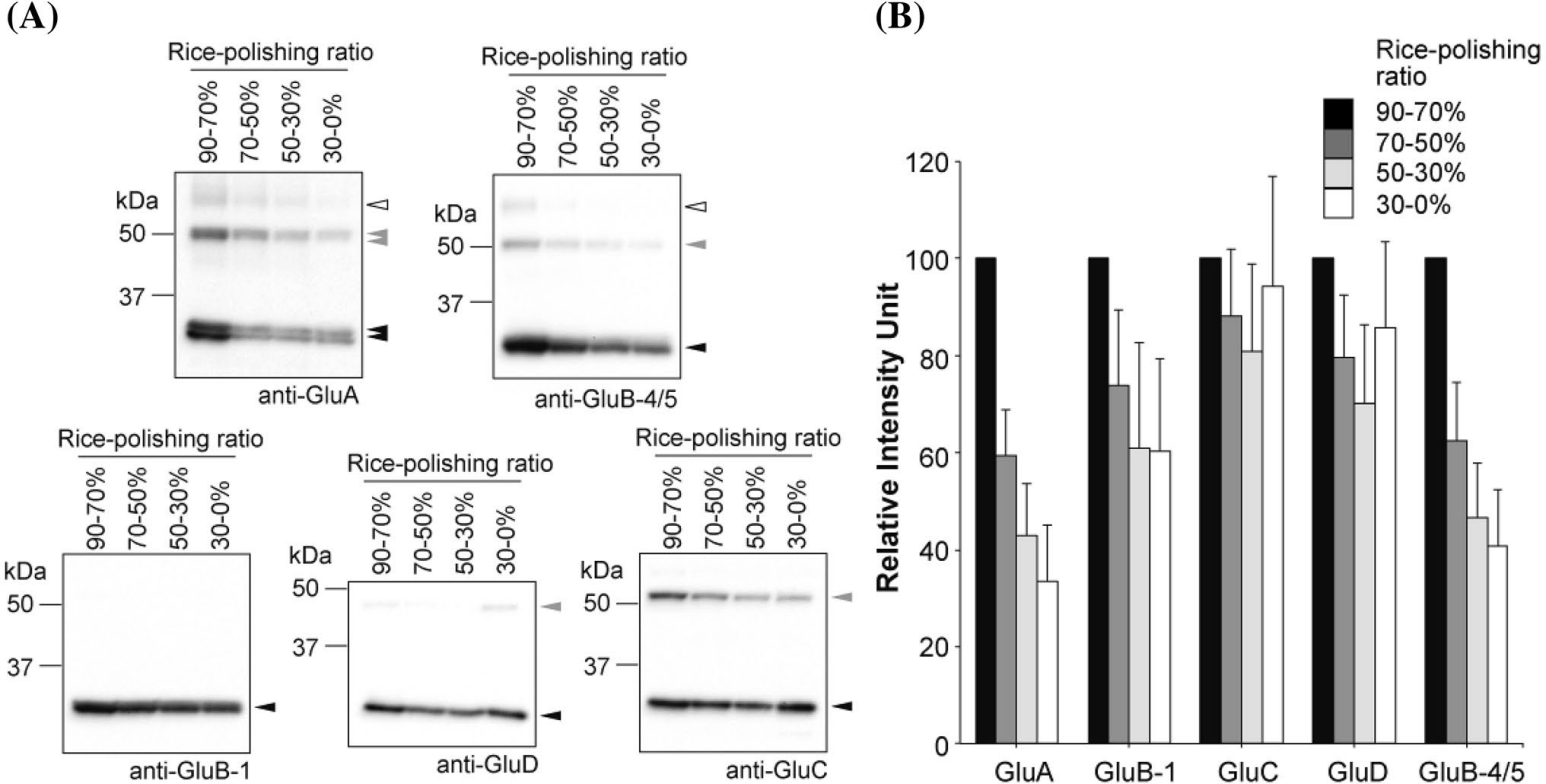
Immunoblot analysis for glutelin subfamilies of fractionated rice powder. **a** Proteins extracted from fractionated rice powder were separated by SDS-PAGE and analyzed by immunoblotting using anti-GluA, anti-GluB-1, anti-GluC, anti-GluD, and anti-GluB-4/5 antibodies. Each lane contains the same weight of rice powder. Black triangles indicate glutelin acidic subunits. Gray triangles indicate immature pro-glutelin. Open triangles indicate a putative dimeric form of the glutelin acidic subunit. A representative result from the *Nipponbare* cultivar harvested in 2011 is shown. **b** Relative intensity of the five glutelin subtype in rice grains. Seven species of *japonica* rice (harvested in 2009, 2010, and 2011) were analyzed (*n* = 14 minimum). The intensity of detected bands corresponding to the fraction with a 90–70% rice-polishing ratio is fixed at 100. Relative intensity is represented with error bars

Subsequently, localization of rice glutelins was analyzed by immunoblotting using fractionated rice powder followed by the quantification of protein bands. Results of immunoblotting of fractionated rice powders from rice grains harvested in 2009, 2010, and 2011 are shown in Supplementary Figs. 2, 3, and 4, respectively. An example of the immunoblotting result of *Nipponbare* rice harvested in 2011 is shown in Fig. 3a. The anti-GluA antibody is expected to cross-react with two GluA subfamily proteins, GluA-1 and GluA-2, because of their epitope similarity. GluA formed doublet bands, particularly when it was electrophoresed for a long time (Fig. 3a). PNGase F treatment did not diminish the doublet band, which suggests that the doublet was not caused by the *N*-glycosylation of GluA (Supplementary Fig. 5). The multiple banding pattern of GluA also has been observed previously (Katsube-Tanaka et al. 2004; Kawakatsu et al. 2008, 2010), and possibly can be attributable to the presence of two proteins. In rice grains, GluA strongly localized in the outer region of the endosperm (Fig. 3). The intensity of the GluA bands diminished markedly when the rice-polishing ratio decreased from 90 to 70 to 70–50% fractions and gradually decreased toward the central region of the rice grain (Fig. 3 and Supplementary Figs. 2, 3, and 4). Glutelin precursors (pro-glutelin) and glutelin acidic subunits were similar in terms of their localizations. Small quantities of the dimeric form of GluA acidic subunit were observed at around 60 kDa. The intensity of GluB-1 and GluB-4/5 bands gradually decreased with polishing. However, more GluB-1 than GluB-4/5 localized in the inner region of the rice grain (Fig. 3), particularly in the *Gohyakumangoku* cultivar (Supplementary Figs. 2, 3, and 4). Contrarily, in the *Dewanosato* cultivar, the signal intensity of GluB-1 markedly diminished with polishing as seen in GluA and GluB-4/5 (Supplementary Figs. 2, 3, and 4). Interestingly, GluC and GluD appeared to localize throughout the endosperm (Fig. 3). Immunoblot analysis identified GluD as the lowest molecular weight glutelin, in agreement with a previous report (Kawakatsu et al. 2008). The GluD localization pattern in mature rice grains was somewhat unique, with this protein being more abundant in the 90–70% fraction, followed by the 30–0%, 70–50% and 50–30% fractions (Fig. 3). Surprisingly, in some cases, the GluC expression level in the inner region was comparable to that in the outer region of the endosperm (Fig. 3). This localization pattern was similar for all years (Supplementary Figs. 2, 3, and 4) and among the rice cultivars examined, thus indicating that the localization patterns are not dramatically affected by temperature during development and are a consistent characteristic of the *japonica* subspecies. In conclusion, the glutelin proteins are found mostly in the outer region of the rice grain, with GluA being the most abundant, followed by GluB-4/5, GluB-1, GluD, and GluC.

### Glutelin protein and mRNA expression in developing rice seeds

**Fig. 4:**
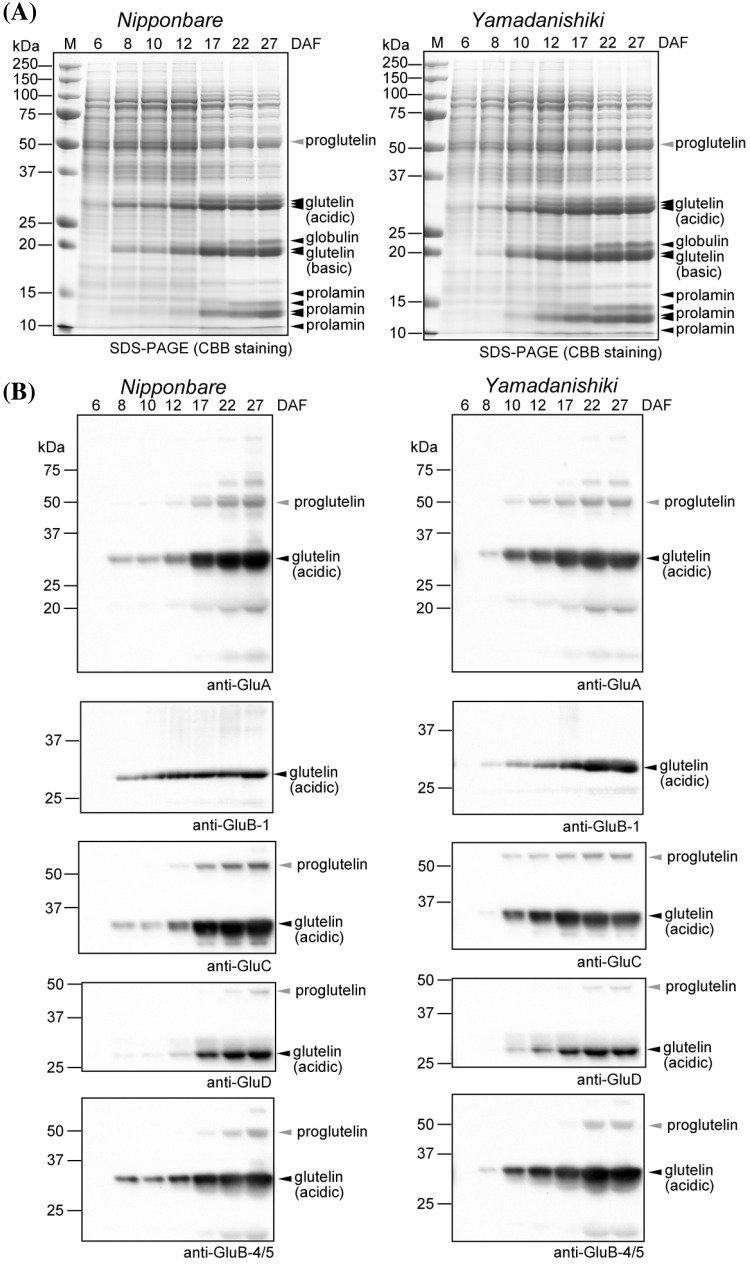
Expression pattern of glutelin subfamilies during rice seed development. **a** CBB staining of total protein in immature seeds of *Nipponbare* and *Yamadanishiki* rice. Each lane contains the same weight of rice grains. M = molecular size marker. Proteins were extracted from immature rice seeds at 6, 8, 10, 12, 17, 22, and 27 DAF. Glutelin acidic subunit, glutelin basic subunit, globulin, and prolamins are indicated by black arrowheads. Pro-glutelin polypeptides are indicated by gray arrowheads. **b** Immunoblot analysis of the five glutelin species. Each lane contains the same weight of rice grains. Glutelin acidic subunits are indicated by black arrowheads. Pro-glutelins are indicated by gray arrowheads

Immunoblot analyses reveal that specific glutelin subtypes vary in their distribution in the rice endosperm. To corroborate these results, we conducted immunofluorescence labeling microscopy analysis using the whole rice grain. To obtain fine paraffin sections of rice grains, we examined the temporal glutelin protein accumulation pattern, because we could not obtain fine immunofluorescence labeling result using mature rice grains. Initially, total protein was visualized by CBB staining in developing *Nipponbare* and *Yamadanishiki* rice seeds at 6, 8, 10, 12, 17, 22, and 27 DAF, as shown in Fig. 4a. As reported previously (Yamagata et al. 1982), glutelins accumulate for several days before prolamins. Glutelins were first detected at 8 DAF (Fig. 4). Immunoblot analysis of each glutelin subfamily clearly showed the expression pattern of glutelins (Fig. 4b), with the maximum glutelin expression level was reached at 17 to 22 DAF in both *Nipponbare* and *Yamadanishiki* cultivars (Fig. 4b). However, there was no substantial difference in the temporal expression patterns of the different glutelin subfamilies (Fig. 4b).

Furthermore, to confirm the temporal mRNA expression patterns of glutelin subfamilies, we performed quantitative real-time PCR in developing rice seeds at 6, 8, 10, 12, and 17 DAF (Supplementary Fig. 6). Maximum mRNA expression of GluA and GluB was attained around 12 DAF, in agreement with previous reports (Onodera et al. 2001; Yamamoto et al. 2006), whereas maximum mRNA expression of GluC (*Nipponbare*) and GluD (Both *Nipponbare* and *Yamadanishiki*) was observed at 17 DAF. Based on these results, we used immature rice seeds at 17 DAF for immunofluorescence labeling assays.

### Immunofluorescence labeling microscopy analysis: lateral sections

**Fig. 5:**
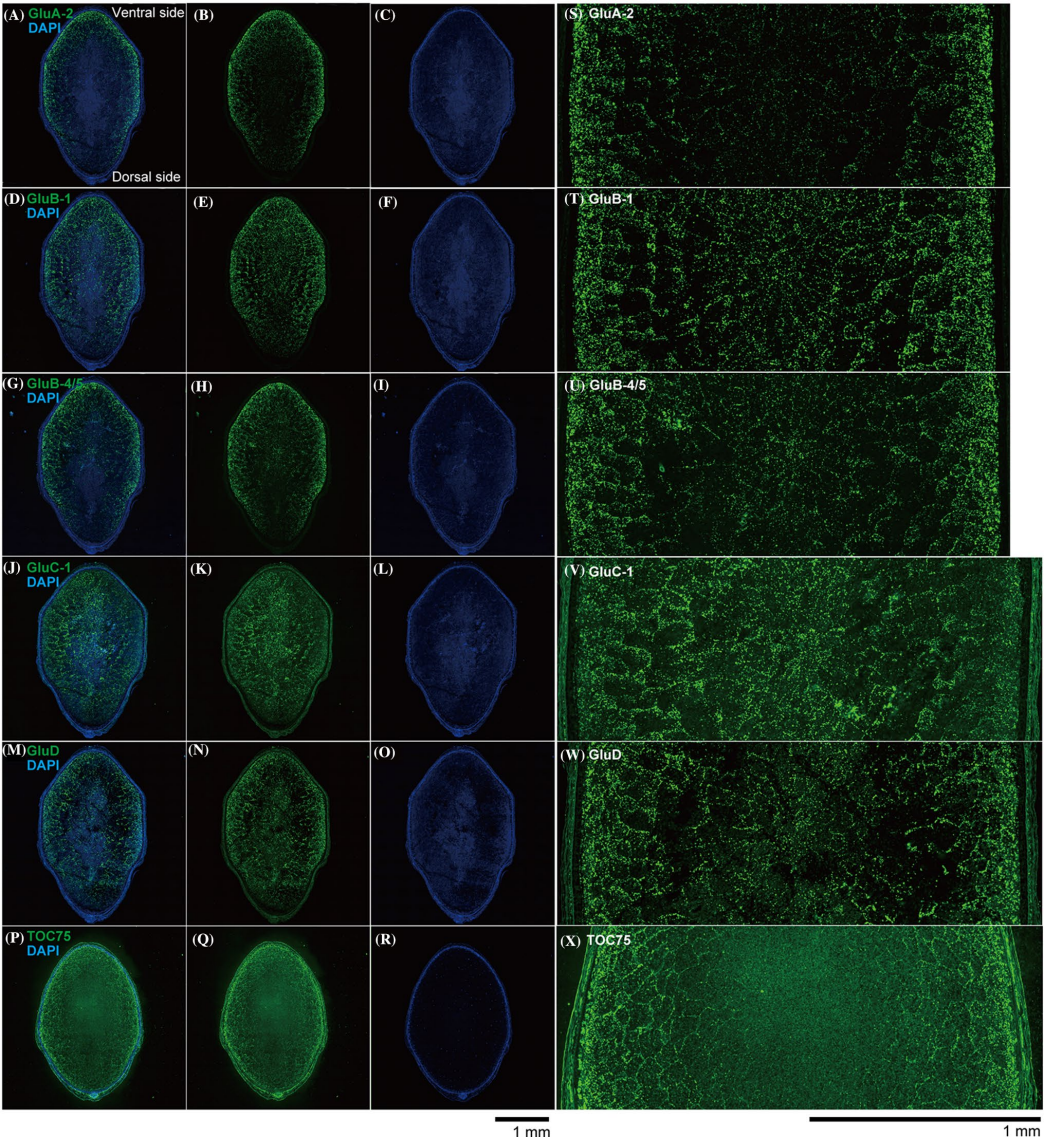
Glutelin subfamily-dependent localization in developing rice seed: lateral sections. Immunofluorescence microscopy images of developing rice seed (17 DAF) of *Yamadanishiki* by immunofluorescence labeling (green fluorescence) with primary antibodies against the five glutelin subfamilies (GluA-2, GluB-1, GluB-4/5, GluC, and GluD) and amyloplast marker protein, TOC75. **a**–**c** Anti-GluA. **d**–**f** Anti-GluB-1. **g**–**i** Anti-GluB-4/5. **j**–**l** Anti-GluC. **m**–**o** Anti-GluD. **p**–**r** Anti-TOC75. Upper and lower sides correspond to the ventral and dorsal sides of the rice grain, respectively. **a, d, g, j, m**, and **p** Merge. **b, e, h, k, n**, and **q** Glutelins and TOC75. **c, f, i, l, o**, and **r** DAPI. **s, t, u, v, w**, and **x** Enlarged images of the central side of rice grains from (**b, e, h, k, n, q)**. Scale bars = 1.0 mm. Four independent experiments were examined and representative data are shown

Lateral sections of developing *Yamadanishiki* rice seeds were treated with antibodies raised against each glutelin subfamily and then detected by green fluorescence (Fig. 5). As shown in Fig. 5, GluA and GluB-4/5 strongly localized in the outer region of the rice grain, indicating that they localize in the outer region of starchy endosperm, including subaleurone layer, although they were weakly found in the inner endosperm (Fig. 5). In starchy endosperm, GluA and GluB-4/5 expression levels gradually diminished from the outer region toward the central region. Although GluB-1 localized to the outer region of the rice grain, a strong fluorescence signal also was detected in the inner endosperm region, suggesting that this protein localized to both regions (Fig. 5). Similar to GluA and GluB-4/5, the GluB-1 expression level gradually diminished from the outer region of starchy endosperm toward the central region (Fig. 5). Unlike GluA, GluB-1, and GluB-4/5, the two subfamilies GluD and GluC tended to localize throughout the bulky endosperm, including the subaleurone layers (Figs. 3, 5). Similar results were observed in developing *Nipponbare* and *Gohyakumangoku* rice grains at 17 DAF (data not shown). The expression levels of all glutelins, particularly GluA, GluB-1, and GluB-4/5, were stronger on the ventral side of grains than on the dorsal side (Fig. 5). TOC75, a 75-kDa amyloplast outer envelope membrane-associated protein in *O. sativa*, was expressed evenly across the entire endosperm in rice grains (Fig. 5).

**Fig. 6:**
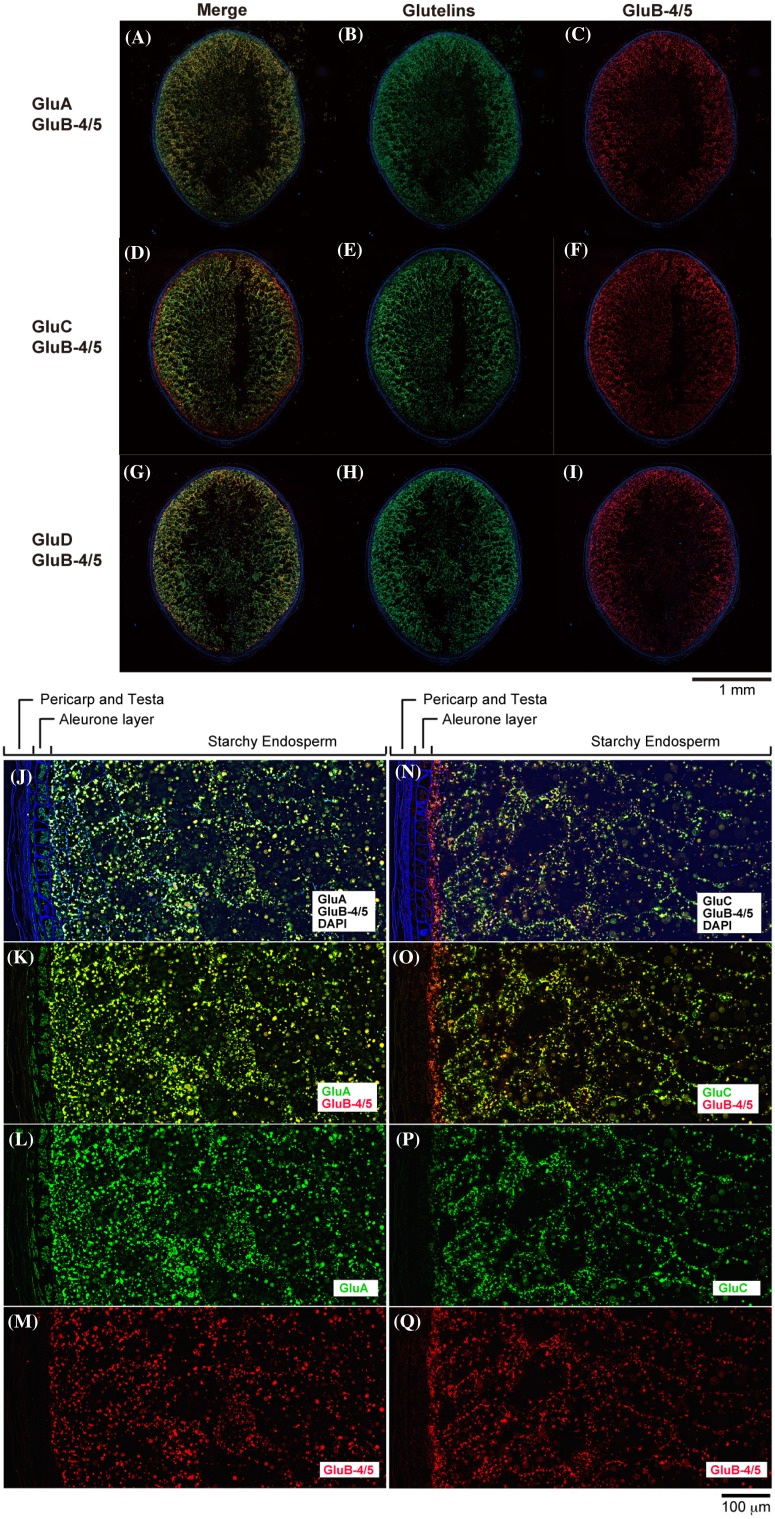
Co-immunofluorescence labeling shows that the localization pattern of GluC is different from GluA in developing rice seed. Co-immunofluorescence microscopy images of the lateral section of developing rice seeds (17 DAF) of *Yamadanishiki* by the immunofluorescence labeling with four glutelin subtype antibodies, GluA, GluC, and GluD (green), and GluB-4/5 (red). **a, d**, and **g** Merge. **b, e**, and **h** GluA, GluC, and GluD, respectively. **c, f**, and **i** GluB-4/5. Upper and lower sides of each (**a**–**i**) correspond to the ventral and dorsal sides of the rice grain, respectively. Scale bar = 1.0 mm. **j**–**m** Enlarged image of the central region of the panel “**a**.” **j** Merge. **k** GluA (Green) and GluB-4/5 (red). **l** GluA. **m** GluB-4/5. **n**–**q** Enlarged image of the central region of the panel “**d**.” **n** Merge. **o**: GluC (Green) and GluB-4/5 (red). **p** GluC. **q** GluB-4/5. Scale bar = 100 µm. Three independent experiments were examined and representative data are shown

To clarify the localization sites of the glutelin subfamilies, co-immunofluorescence labeling microscopy analysis was performed on a combination of GluB-4/5 and the other glutelin subfamilies. As shown in Fig. 6a-c, GluA and GluB-4/5 co-localized mainly in the outer endosperm region of rice grain. GluD expression was considerably stronger in the central endosperm than that of GluB-4/5 (Fig. 6g–i), indicating that GluD expression in the central endosperm is considerably greater than that of GluA and GluB-4/5. Finally, GluC expression was quite different from that of GluB-4/5 (Fig. 6d–f), as it were absent in the outermost of the subaleurone layer, if any, but present in the central starchy endosperm.

Higher magnification images of co-immunofluorescence labeling microscopy analysis of combined GluA with GluB-4/5 (Fig. 6j–m) revealed that both localized primarily in the subaleurone layer and the outer starchy endosperm region of rice grain. However, co-immunofluorescence labeling microscopy analysis images of combined GluC with GluB-4/5 (Fig. 6n–q) revealed that GluC did not co-localize with GluA in the outermost endosperm region.

### Immunofluorescence labeling microscopy analysis: vertical sections

**Fig. 7:**
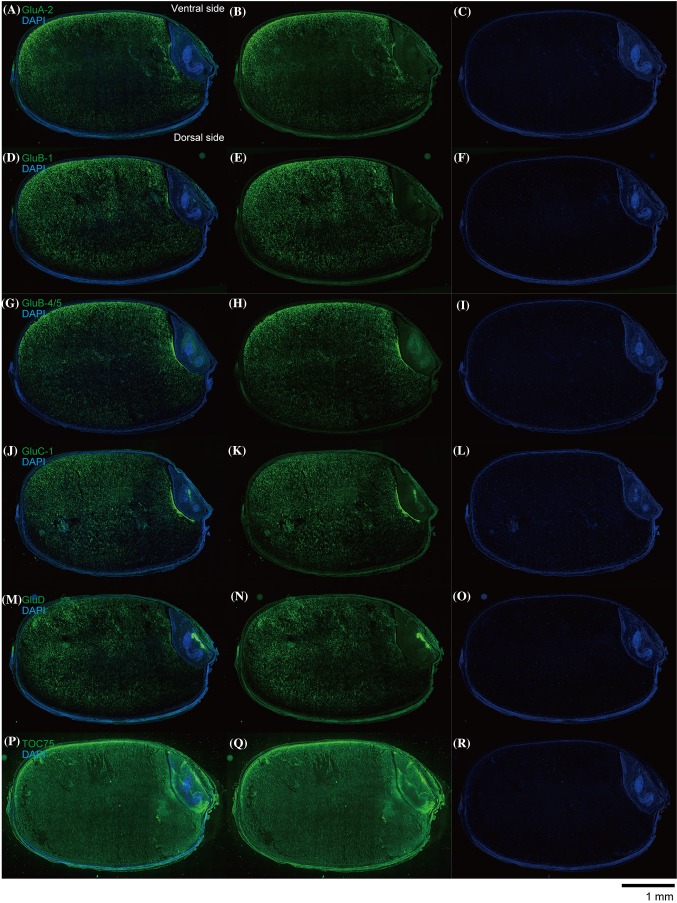
Glutelin subfamily-dependent localization in developing rice seed: vertical sections. Immunofluorescence microscopy images of developing rice seed (17 DAF) of *Yamadanishiki* by immunofluorescence labeling (green fluorescence) with primary antibodies against the five glutelin subfamilies (GluA-2, GluB-1, GluB-4/5, GluC, and GluD) and amyloplast marker protein, TOC75. **a**–**c** Anti-GluA. **d**–**f** Anti-GluB-1. **g**–**i** Anti-GluB-4/5. **j**–**l** Anti-GluC. **m**–**o** Anti-GluD. **p**–**r** Anti-TOC75. Upper and lower sides correspond to the ventral and dorsal sides of the rice grain, respectively. **a, d, g, j, m**, and **p** Merge. **b, e, h, k, n**, and **q** Glutelins and TOC75. **c, f, i, l, o**, and **r** DAPI. Scale bar = 1.0 mm. Four independent experiments were examined and representative data are shown

To investigate localization patterns of the glutelin subfamily in vertical sections of the rice grain, we performed immunofluorescence labeling microscopy analysis of developing *Yamadanishiki* rice grains. As shown in Fig. 7a–i, strong signals were detected in the outer endosperm region, including the subaleurone layer, on the ventral side of rice grains, but weak signal emerged on the dorsal side in GluA, GluB-1, and GluB-4/5, corresponding to the results in lateral sections (Fig. 5). In contrast, GluC and GluD localized to the inner starchy endosperm rather than the subaleurone layer (Fig. 7j–o). GluC and GluD localized to the outer endosperm region on the ventral side of rice grains compared with the dorsal side, but significant amounts were detected in the inner endosperm region (Fig. 7j–o), consistent with the results of the immunofluorescence labeling analyses in lateral sections. Unexpectedly, glutelin (GluB-4/5, GluC, and GluD) signals were also detected on the boundary between the endosperm and embryo (close to scutellum) (Fig. 7h, k, n). As expected, TOC75 was expressed evenly in the whole endosperm of the rice grain (Fig. 7p–r).

**Fig. 8:**
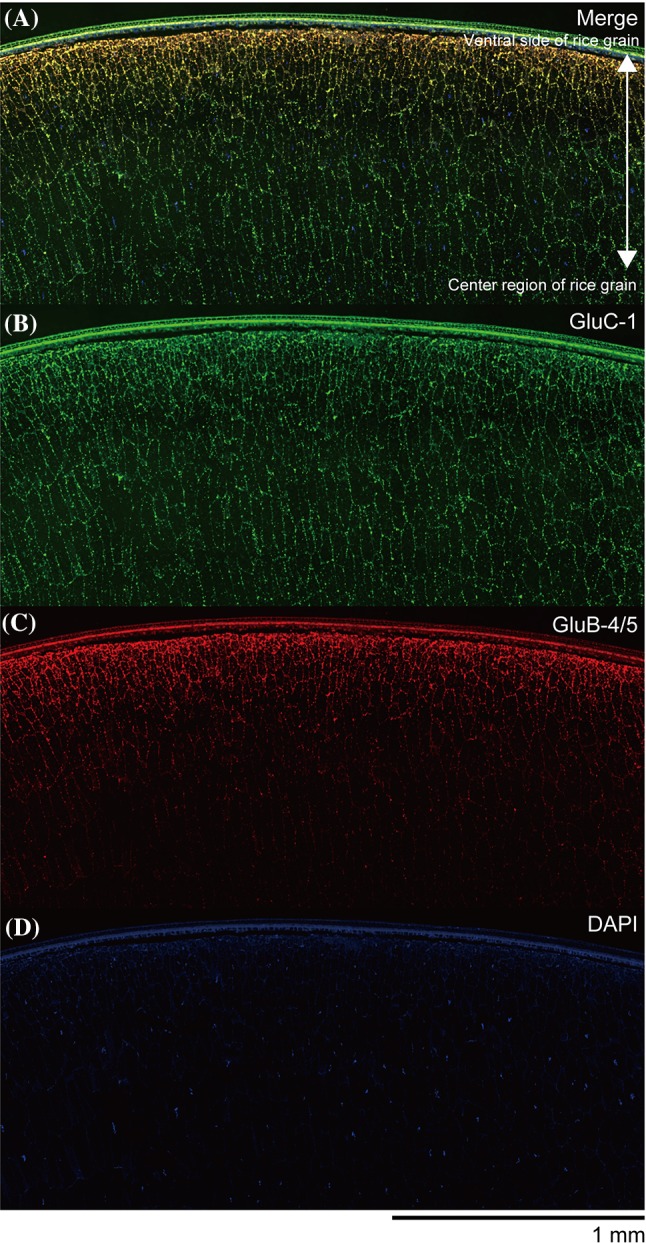
Enlarged co-immunofluorescence microscopy image of glutelin at the center of the ventral region in developing rice seed: vertical sections. Co-immunofluorescence microscopy images of a vertical section of the developing *Yamadanishiki* seed (17 DAF) by immunofluorescence labeling with anti-GluC antibody (green) and GluB-4/5 antibody (red). The center of the ventral region of the rice grain is enlarged. **a** Merge. **b** GluC. **c** GluB-4/5. **d** DAPI. Scale bar = 1.0 mm. Three independent experiments were examined and representative data are shown

Co-immunofluorescence labeling microscopy analysis of combined GluB-4/5 with GluC clearly revealed that GluB-4/5 strongly located to the subaleurone layer and the outer starchy endosperm region of the rice grain, whereas the GluC signal was detected in the inner and outer endosperm regions of the rice grain (Fig. 8). Unexpectedly, remarkable glutelin signals were also found in the aleurone layer, testa, pericarp, and embryo in the rice grain (Figs. 7, 8).

### Most glutelins are not expressed in the embryo, and aleurone layer in rice grain

**Fig. 9:**
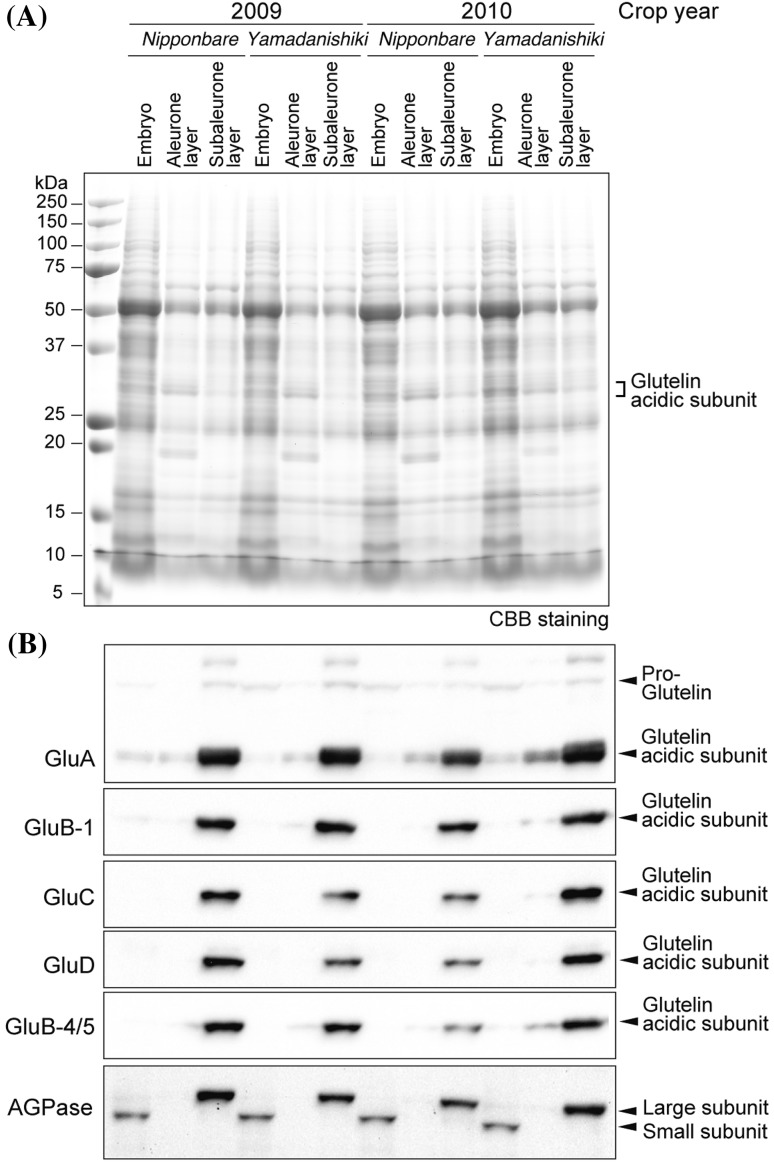
Expression level of glutelins in the aleurone layer fraction, the subaleurone fraction, and the embryo in mature rice grains. Proteins extracted and separated by SDS-PAGE from the aleurone layer fraction, the subaleurone layer fraction, and the embryo of *Nipponbare* and *Yamadanishiki*, which were harvested in 2009 and 2010. **a** Total proteins in the aleurone layer fraction, the subaleurone fraction, and the embryo by CBB staining. Each lane contains the same weight of tissue. **b** Immunoblot analysis against glutelins and ADP-glucose pyrophosphorylase (AGPase). A pro-glutelin band was detected only with the anti-GluA antibody. Each lane contains the same weight of tissue. Two independent experiments were examined and typical data are shown

Immunofluorescence labeling microscopy analysis using glutelin antibodies suggests the possibility of glutelins being expressed in vegetative organs, such as pericarp and testa other than in the starchy endosperm in rice grains. To investigate whether glutelin subtypes are expressed in the embryo, aleurone layer, pericarp, and testa, immunoblot analysis was performed in mature rice grains separated into the aleurone layer fraction containing pericarp and testa, the subaleurone fraction, and the embryo. These fractions contained a different pattern of ADP-glucose pyrophosphorylase (AGPase) large and small subunits, indicating successful tissue separation (Fig. 9). All five glutelin subtypes were expressed in the subaleurone layer fraction (Fig. 9), although the intensity in this layer was considerably lower than that of the 50–0% fractions of starchy endosperm (Supplementary Fig. 7). Among the glutelin subfamilies, only small amounts of GluA were detected ectopically in the embryo (Fig. 9), as the proportion of the pro-glutelin form of GluA was much higher than the GluA acidic subunit. The abundance of pro-glutelin in the embryo can be attributed to the expression level of a vacuole-type aspartic protease (Wang et al. 2009; Kumamaru et al. 2010). By contrast, the other glutelins were not detected in the embryo fraction. GluA and GluB-4/5, which primarily expressed in the outer endosperm region, tended to be marginally detected in the aleurone layer fraction, but other glutelin subtypes were hardly detected in the aleurone layer fraction.

## Discussion

This study investigated the patterns of glutelin subtype localization using subfamily-specific antibodies in seven types of *japonica* rice grain cultivars across 3 years. Strong GluA (GluA-1 and GluA-2) signals were detected in the outer region of the endosperm, including the subaleurone layer. GluA also was found at lower levels in the embryo, whereas no signal was detected for other glutelin subfamilies (Fig. 9). Regulation of GluA gene expression can differ slightly from those of the other glutelin subfamilies. Previously, colorimetric assays using promoters of the *GluA-1, GluA-2*, and *GluA-3* genes fused to the GUS reporter gene in a developing rice grain revealed that the *GluA* promoter activity was stronger in the outer part but weaker in the inner part of the endosperm (Qu et al. 2008). In this regard, our findings in this study corresponded to the previous study. Additionally, we revealed that the expression of GluA (GluA-1 and GluA-2) also was much higher on the ventral side than on the dorsal (abaxial) side (Figs. 5, 7) for the first time.

The localization pattern of GluB-4/5 in the endosperm was similar to that of GluA, with strong localization in the outer regions and a gradual decrease toward the central region. The localization pattern of GluB-1 was similar to that of GluB-4/5, but there was a moderate signal of GluB-1 in the inner region of the endosperm. The cultivar-specific localization pattern of GluB-1 in *Gohyakumangoku* was observed using an immunoblot assay (Supplementary Figs. 2, 3, 4), which showed a relatively constant level of GluB-1 expression in the starchy endosperm. The results of immunofluorescence labeling microscopy analysis of GluB-1 for *Gohyakumangoku* support the immunoblot data (data not shown). Previously, it has been shown that the full-length *GluB-1* promoter had strong activity in the aleurone and subaleurone layers, but was weakly activated in the center of the endosperm (Wu et al. 1998). Using the same method, Qu and Takaiwa showed that *GluB-1, GluB-2*, and *GluB-4* gene promoters were activated in the outer endosperm in a specific manner (Qu and Takaiwa 2004). The previous finding, with regard to the spatial expression pattern of GluB-1 and GluB-4, supports our findings. However, another previous report indicating that *GluB-5* promoter directed GUS expression in the whole endosperm at 17 DAF (Qu et al. 2008) does not completely coincide with our findings. This may be attributable to differences in the resolution limits of the methods used in each experiment.

Interestingly, the GluC and GluD localization profiles differed from those of GluA and GluB subfamilies (Fig. 3, 5, 6, 7, and 8). Both GluC and GluD localized throughout the endosperm, in rough agreement with previous reports (Kawakatsu et al. 2008; Qu et al. 2008). However, the expression levels of all glutelins, particularly GluA, GluB-1 and GluB-4/5, were stronger on the ventral side than on the dorsal (abaxial) side (Figs. 5, 7). Because the total proteins in rice seeds gradually decreased from the outer region toward the central region of the endosperm (Fig. 2b), both GluC and GluD, at the least, are not the major glutelin proteins.

Expression of GluA and GluB mRNAs differed slightly from that of GluC and GluD (Supplementary Fig. 6). The difference in temporal mRNA expression patterns can be linked to the spatial patterns of glutelin expression. Glutelin mRNA expression is positively regulated by two transcription factors, Rice Prolamin Box binding Factor (RPBF) and RIce Seed Beta-Zipper (RISBZ)1 (Onodera et al. 2001; Yamamoto et al. 2006; Kawakatsu et al. 2009). RPBF and RISBZ1 are specifically expressed in the endosperm region, including the aleurone and subaleurone layers (Onodera et al. 2001; Yamamoto et al. 2006), and, with RISBZ1 expression mainly confined to the aleurone and subaleurone layers (Onodera et al. 2001). These transcription factors operate synergistically and are important for normal development of the aleurone layer and grain filling through positive expression of seed storage proteins and starch synthesis (Onodera et al. 2001; Yamamoto et al. 2006; Kawakatsu et al. 2009). The expression levels of RPBF and RISBZ1 in developing rice seeds have been reported to reach a maximum before 15 DAF (Onodera et al. 2001; Yamamoto et al. 2006), however their expression timing is much earlier in RISBZ1 (5 DAF) than in RPBF. Knock down of RPBF in rice grains markedly diminished *GluD* mRNA expression in rice seeds at 14 DAF and thoroughly abolished GluD protein expression in mature rice seeds; however, only a limited effect was observed with *GluA* and *GluB* mRNA and protein levels (Kawakatsu et al. 2009). In our study, considerable expression of GluD was observed in the outer and central regions of the endosperm (Figs. 3, 5). GluD expression could be controlled by RPBF or unknown transcription factors in whole grains, including the endosperm, whereas RISBZ1 or unknown transcription factors could contribute to GluD expression in the outer region of rice grains. The GluC localization pattern is unique among glutelin subfamilies, particularly as this protein is evenly expressed across the entire endosperm in rice grains (Figs. 3, 5, 7). Previously, promoter analyses revealed that the *GluC* promoter does not contain any sequences of the GCN4 motif “TGA(G/C)TCA” that binds to RISBZ1, the prolamin box “TGTAAAG” that binds to RPBF, and one of the major endosperm specificity-determining motifs (the AACA motif), although it contains the ACGT motif (Qu et al. 2008). The *cis*-elements on the *GluC* promoter could be one of the responsible factors controlling its localization in rice grains. Some inhibitory effects on glutelin expression via RPBF and RISBZ1 in developing rice seeds have been shown to be a result of the CCCH-type zinc finger protein OsGZF1 (Chen et al. 2013) and salt-responsive ERF1 (SERF1) (Schmidt et al. 2014). OsGZF1 is expressed in the early stages of rice seed development (5–10 DAF) and directly binds to the *GluB-1* promoter region, which results in the inhibition of RISBZ1-dependent induction of GluB-1 (Chen et al. 2013). SERF1 knockout or knockdown increased *RPBF* and *RISBZ1* mRNA expression in the early stages of rice seed development, resulting in increased glutelin mRNA expression, as well as increased expression of starch synthesis genes (Schmidt et al. 2014). In a colorimetric promoter activity assay of using GUS reporter, the promoter activity is affected by the glutelin promoter length used as well as untranslated nucleotide sequences. Li et al. suggested that nucleotide sequences of 3′-untranslated regions (UTRs) in the glutelin mRNA could be involved in localization patterns of their proteins in the rice endosperm and associated with the intensity of protein expression levels by increasing the mRNA levels (Li et al. 2012). Liu et al. showed that the glutelin 5′-UTR conferred increased translational efficiency, but did not change mRNA levels (Liu et al. 2010). These data indicate that promoter activity assay only itself is not sufficient for reliable evaluation of protein localization in rice grain. Protein localization analyses using glutelin subtype-specific antibodies demonstrated in this study provides reliable results and new insight into glutelin localization in rice grain. Together, temporal and spatial molecular orchestration of DNA-binding proteins as well as *cis*-elements on the glutelin genes/promoters could well be the definitive factors controlling glutelin localization and intensity in rice grains although further research is required.

Immunofluorescence labeling microscopy analysis showed a stronger glutelin signal on the boundary between endosperm and embryo (Fig. 7). This observation is similar to a previous report of the GluB family using a GUS assay of glutelin promoters (Qu and Takaiwa 2004). It is well known that endosperm is an important nutrient source that contributes to the germination of mature rice seeds (Day et al. 2008; Zhou et al. 2013). Glutelins localized around scutellum may be preferentially digested, and the digested products may be provided in the early stage of embryo germination process. Observation by the methodology of immunofluorescence assay might be another reason explaining the glutelin detection on the boundary between endosperm, as well as on the embryo, in the aleurone layer, and the pericarp and testa. In our immunofluorescence assay, we either activated the thin layer paraffin-embedded rice grain by slightly heating it or did not activate it before reaction with the first antibody to avoid gelatinization of starch or cracking/breaking of the tissue. Depending on the plant tissue type, non-specific adsorption of the antibodies to vegetative tissues can occur. We sometimes detected glutelins and TOC75 in vegetative tissues, such as the aleurone layer, the pericarp and the testa (Figs. 7, 8). This tendency can be found in another report (Wang et al. 2018). Considering that glutelins were not detected clearly in the aleurone layer fraction containing the pericarp and testa by immunoblotting analysis in mature rice seeds (Fig. 9), most glutelins may not be expressed in the aleurone layer, much less the pericarp and testa in rice grains. Of note, when glutelins were detected on the boundary between the endosperm and embryo, they also tended to be detected in the inner region of endosperm by immunofluorescence labeling assay (data not shown).

In summary, we used biochemical and cell biological approaches to demonstrate glutelin subtype-dependent localization in rice grains. These two independent approaches provided consistent evidence for differences in glutelin subtype localization patterns. Our findings and discussion in this research will be fundamental for future studies of rice protein research and will be applicable to other cereal protein research. Further, these findings can contribute to the fields of rice breeding science, food science, and plant physiology.

## Electronic supplementary material

Below is the link to the electronic supplementary material.

Supplementary material 1 (DOCX 18 KB)

Supplementary material 2 (DOCX 4243 KB)

Supplementary material 3 (DOCX 14 KB)

## Abbreviations

ER: Endoplasmic reticulum
PB: Protein body
PSV: Protein storage vacuole
GUS: β-glucuronidase
DAF: Days after flowering
RAP: Rice annotation project
SDS-PAGE: Sodium dodecyl sulfate-poly acrylamide gel electrophoresis
CBB: Coomassie brilliant blue
PCR: Polymerase chain reaction

## References

[CR1] Ashida K, Araki E, Maruyama-Funatsuki W, Fujimoto H, Ikegami M (2013). Temperature during grain ripening affects the ratio of type-II/type-I protein body and starch pasting properties of rice (*Oryza sativa* L.). J Cereal.

[CR2] Chen Y, Sun A, Wang M, Zhu Z, Ouwerkerk PB (2013). Functions of the CCCH type zinc finger protein OsGZF1 in regulation of the seed storage protein GluB-1 from rice. Plant Mol Biol.

[CR3] Day RC, Herridge RP, Ambrose BA, Macknight RC (2008). Transcriptome analysis of proliferating Arabidopsis endosperm reveals biological implications for the control of syncytial division, cytokinin signaling, and gene expression regulation. Plant Physiol.

[CR4] Fukuda M, Satoh-Cruz M, Wen L, Crofts AJ, Sugino A, Washida H, Okita TW, Ogawa M, Kawagoe Y, Maeshima M, Kumamaru T (2011). The small GTPase Rab5a is essential for intracellular transport of proglutelin from the Golgi apparatus to the protein storage vacuole and endosomal membrane organization in developing rice endosperm. Plant Physiol.

[CR5] Fukuda M, Wen L, Satoh-Cruz M, Kawagoe Y, Nagamura Y, Okita TW, Washida H, Sugino A, Ishino S, Ishino Y, Ogawa M, Sunada M, Ueda T, Kumamaru T (2013). A guanine nucleotide exchange factor for Rab5 proteins is essential for intracellular transport of the proglutelin from the Golgi apparatus to the protein storage vacuole in rice endosperm. Plant Physiol.

[CR6] Juliano BO (1993) Rice in human nutrition. Published with the collaboration of the international rice research institute food and agriculture organization of the united nations, Rome

[CR7] Kanauchi M (2013) SAKE alcoholic beverage production in Japanese food industry. Food industry innocenzo Muzzalupo. IntechOpen. 10.5772/53153

[CR8] Katsube-Tanaka T, Duldulao JB, Kimura Y, Iida S, Yamaguchi T, Nakano J, Utsumi S (2004). The two subfamilies of rice glutelin differ in both primary and higher-order structures. Biochim Biophys Acta.

[CR9] Katsube-Tanaka T, Iida S, Yamaguchi T, Nakano J (2010). Capillary electrophoresis for analysis of microheterogeneous glutelin subunits in rice (*Oryza sativa* L.). Electrophoresis.

[CR10] Kawagoe Y, Suzuki K, Tasaki M, Yasuda H, Akagi K, Katoh E, Nishizawa NK, Ogawa M, Takaiwa F (2005). The critical role of disulfide bond formation in protein sorting in the endosperm of rice. Plant Cell.

[CR11] Kawakatsu T, Takaiwa F (2010). Cereal seed storage protein synthesis: fundamental processes for recombinant protein production in cereal grains. Plant Biotechnol J.

[CR12] Kawakatsu T, Yamamoto MP, Hirose S, Yano M, Takaiwa F (2008). Characterization of a new rice glutelin gene GluD-1 expressed in the starchy endosperm. J Exp Bot.

[CR13] Kawakatsu T, Yamamoto MP, Touno SM, Yasuda H, Takaiwa F (2009). Compensation and interaction between RISBZ1 and RPBF during grain filling in rice. Plant J.

[CR14] Kawakatsu T, Hirose S, Yasuda H, Takaiwa F (2010). Reducing rice seed storage protein accumulation leads to changes in nutrient quality and storage organelle formation. Plant Physiol.

[CR15] Kitagaki H, Takagi H (2013). Mitochondrial metabolism and stress response of yeast: applications in fermentation technologies. J Biosci Bioeng.

[CR16] Kumamaru T, Uemura Y, Inoue Y, Takemoto Y, Siddiqui SU, Ogawa M, Hara-Nishimura I, Satoh H (2010). Vacuolar processing enzyme plays an essential role in the crystalline structure of glutelin in rice seed. Plant Cell Physiol.

[CR17] Kusaba M, Miyahara K, Iida S, Fukuoka H, Takano T, Sassa H, Nishimura M, Nishio T (2003). Low glutelin content1: a dominant mutation that suppresses the glutelin multigene family via RNA silencing in rice. Plant Cell.

[CR18] Li WJ, Dai LL, Chai ZJ, Yin ZJ, Qu LQ (2012). Evaluation of seed storage protein gene 3′-untranslated regions in enhancing gene expression in transgenic rice seed. Transgenic Res.

[CR19] Lin CJ, Li CY, Lin SK, Yang FH, Huang JJ, Liu YH, Lur HS (2010). Influence of high temperature during grain filling on the accumulation of storage proteins and grain quality in rice (*Oryza sativa* L.). J Agric Food Chem.

[CR20] Liu WX, Liu HL, Chai ZJ, Xu XP, Song YR, Qu LQ (2010). Evaluation of seed storage-protein gene 5′ untranslated regions in enhancing gene expression in transgenic rice seed. Theor Appl Genet.

[CR21] Masumura T, Kidzu K, Sugiyama Y, Mitsukawa N, Hibino T, Tanaka K, Fujii S (1989). Nucleotide sequence of a cDNA encoding a major rice glutelin. Plant Mol Biol.

[CR22] Mitsukawa N, Hayashi H, Yamamoto K, Kidzu K, Konishi R, Masumura T, Tanaka K (1998). Molecular cloning of a novel glutelin cDNA from rice seeds. Plant Biotechnol.

[CR23] Motoyama T, Maruyama N, Amari Y, Kobayashi K, Washida H, Higasa T, Takaiwa F, Utsumi S (2009). {alpha}’ Subunit of soybean {beta}-conglycinin forms complex with rice glutelin via a disulphide bond in transgenic rice seeds. J Exp Bot.

[CR24] Ohdaira Y, Masumura T, Nakatsuka N, Shigemitsu T, Saito Y, Sasaki R (2011). Analysis of storage protein distribution in rice grain of seed-protein mutant cultivars by immunofluorescence microscopy. Plant Prod Sci.

[CR25] Okuda M, Miyamoto M, Joyo M, Takahashi K, Goto-Yamamoto N, Iida S, Ishii T (2016). The relationship between rice protein composition and nitrogen compounds in sake. J Biosci Bioeng.

[CR26] Onodera Y, Suzuki A, Wu CY, Washida H, Takaiwa F (2001). A rice functional transcriptional activator, RISBZ1, responsible for endosperm-specific expression of storage protein genes through GCN4 motif. J Biol Chem.

[CR27] Qu LQ, Takaiwa F (2004). Evaluation of tissue specificity and expression strength of rice seed component gene promoters in transgenic rice. Plant Biotechnol J.

[CR28] Qu LQ, Wei L, Satoh H, Kumamaru T, Ogawa M, Takaiwa F (2002). Inheritance of alleles for glutelin alpha-2 subunit genes in rice and identification of their corresponding cDNA clone. Theor Appl Genet.

[CR29] Qu LQ, Xing YP, Liu WX, Xu XP, Song YR (2008). Expression pattern and activity of six glutelin gene promoters in transgenic rice. J Exp Bot.

[CR30] Schmidt R, Schippers JH, Mieulet D, Watanabe M, Hoefgen R, Guiderdoni E, Mueller-Roeber B (2014). SALT-RESPONSIVE ERF1 is a negative regulator of grain filling and gibberellin-mediated seedling establishment in rice. Mol Plant.

[CR31] Song Y-J, Choi I-Y, Sharma PK, Kang C-H (2012). Effect of different nitrogen doses on the storage proteins and palatability of rice grains of primary and secondary rachis branches. Plant Prod Sci.

[CR32] Takahashi K, Kohno H (2016). Different polar metabolites and protein profiles between high- and low-quality Japanese Ginjo Sake. PLoS ONE.

[CR33] Takahashi K, Tokuoka M, Kohno H, Sawamura N, Myoken Y, Mizuno A (2012). Comprehensive analysis of dipeptides in alcoholic beverages by tag-based separation and determination using liquid chromatography/electrospray ionization tandem mass spectrometry and quadrupole-time-of-flight mass spectrometry. J Chromatogr A.

[CR34] Takahashi K, Kabashima F, Tsuchiya F (2016). Comprehensive two-dimensional gas chromatography coupled with time-of-flight mass spectrometry reveals the correlation between chemical compounds in Japanese sake and its organoleptic properties. J Biosci Bioeng.

[CR35] Takaiwa F, Oono K (1991). Genomic DNA sequences of two new genes for new storage protein glutelin in rice. Jpn J Genet.

[CR36] Takaiwa F, Kikuchi S, Oono K (1987). A rice glutelin gene family—a major type of glutelin mRNAs can be divided into two classes. Mol Genet Genom.

[CR37] Takaiwa F, Oono K, Wing D, Kato A (1991). Sequence of three members and expression of a new major subfamily of glutelin genes from rice. Plant Mol Biol.

[CR38] Tanaka K, Sugimoto T, Ogawa M, Kasai Z (1980). Isolation and characterization of two types of protein bodies in the rice endosperm. Agric Biol Chem.

[CR39] Tian L, Dai LL, Yin ZJ, Fukuda M, Kumamaru T, Dong XB, Xu XP, Qu LQ (2013). Small GTPase Sar1 is crucial for proglutelin and alpha-globulin export from the endoplasmic reticulum in rice endosperm. J Exp Bot.

[CR40] Wang Y, Zhu S, Liu S, Jiang L, Chen L, Ren Y, Han X, Liu F, Ji S, Liu X, Wan J (2009). The vacuolar processing enzyme OsVPE1 is required for efficient glutelin processing in rice. Plant J.

[CR41] Wang J, Hu P, Lin L, Chen Z, Liu Q, Wei C (2018). Gradually decreasing starch branching enzyme expression is responsible for the formation of heterogeneous starch granules. Plant Physiol.

[CR42] Wing RA, Purugganan MD, Zhang Q (2018). The rice genome revolution: from an ancient grain to green super rice. NatRev Genet.

[CR43] Wu CY, Suzuki A, Washida H, Takaiwa F (1998). The GCN4 motif in a rice glutelin gene is essential for endosperm-specific gene expression and is activated by Opaque-2 in transgenic rice plants. Plant J.

[CR44] Wu X, Liu J, Li D, Liu CM (2016). Rice caryopsis development II: dynamic changes in the endosperm. J Integr Plant Biol.

[CR45] Yamagata H, Tanaka K (1986). The site of synthesis and accumulation of rice storage proteins. Plant Cell Physiol.

[CR46] Yamagata H, Sugimoto T, Tanaka K, Kasai Z (1982). Biosynthesis of storage proteins in developing rice seeds. Plant Physiol.

[CR47] Yamakawa H, Hirose T, Kuroda M, Yamaguchi T (2007). Comprehensive expression profiling of rice grain filling-related genes under high temperature using DNA microarray. Plant Physiol.

[CR48] Yamamoto MP, Onodera Y, Touno SM, Takaiwa F (2006). Synergism between RPBF Dof and RISBZ1 bZIP activators in the regulation of rice seed expression genes. Plant Physiol.

[CR49] Zhou SR, Yin LL, Xue HW (2013). Functional genomics based understanding of rice endosperm development. Curr Opin Plant Biol.

