# Supplementary material for: Glutelin subtype-dependent protein localization in rice grain evidenced by immunodetection analyses
Source: Plant Mol Biol. 2019 Mar 25;100(3):231–46. doi: 10.1007/s11103-019-00855-5 (PMC6542783; doi:10.1007/s11103-019-00855-5)
Supplement: Supplementary file 1 — Supplementary material 1 (DOCX 18 KB) [file 11103_2019_855_MOESM1_ESM.docx]

| Supplementary Table S1. Rice genes, primer sequences, and TaqMan® probe sets used for quantitative real-time PCR. | | | | | | | |
| --- | --- | --- | --- | --- | --- | --- | --- |
| No. | Target Gene | Name | Forward primer | Reverse primer | Reporter 1 Sequence | Accession No. | TaqMan® probe set |
| 1 | Glutelin A | GluA |  |  |  | Os01g0762500 | Os03639831_s1^a^ |
| 2 | Glutelin B | GluB | AACGCCAATCAGCTTGAACCTA | TTGTTGAGCCCGATTGTTGTTG | CCGGCTAATAGGAACTCCTTT | Os02g0249900 | AIHSPCX^b^ |
| 3 | Glutelin C | GluC |  |  |  |  | Os03622704_g1^a^ |
| 4 | Glutelin D | GluD |  |  |  |  | Os03474309_g1^a^ |
| 5 | 17S rRNA | 17S rRNA | CCTTCGGGATCGGAGTAATGATTAA | CTTTCGCAGTTGTTCGTCTTTCATA | TAGTCAGAGGTGAAATTC | X00755 | AID1UT9^b^ |
| ^a^ TaqMan® Gene Expression Assays | | | | | | | |
| ^b^ Custom TaqMan® Gene Expression Assays | | | | | | | |

| Supplementary Table S2. Average temperature (˚C) after rice heading of rice cultivar used in this study. | | | | | | | | | |
| --- | --- | --- | --- | --- | --- | --- | --- | --- | --- |
|  |  |  | Year | | | | | | |
|  |  |  | 2009 | 2010 | 2011 |  | 2009 | 2010 | 2011 |
| Cultivar | Locations |  | during 1-30 days | | |  | during 11-20 days | | |
| *Yamadanishiki* | Hiroshima |  | 21.6 | 26.2 | 24.4 |  | 22.9 | 26.9 | 24.0 |
| *Nipponbare* | Hiroshima |  | 23.8 | 27.4 | 24.9 |  | 23.1 | 27.7 | 24.8 |
| *Gohyakumangoku* | Fukushima |  | 23.3 | 26.6 | 24.6 |  | 23.8 | 26.9 | 26.5 |
| *Koshihikari* | Chiba |  | 26.1 | 28.8 | 27.0 |  | 26.4 | 28.5 | 26.9 |
| *Dewasansan* | Yamagata |  | 23.6 | 27.8 | 26.0 |  | 24.1 | 27.1 | 25.4 |
| *Dewanosato* | Yamagata |  | 21.3 | 26.3 | 24.0 |  | 20.5 | 26.6 | 23.2 |
| *Yumenokaori* | Fukushima |  | 22.9 | 26.8 | 24.9 |  | 23.1 | 26.2 | 24.0 |
