# Supplementary material for: Glutelin subtype-dependent protein localization in rice grain evidenced by immunodetection analyses
Source: Plant Mol Biol. 2019 Mar 25;100(3):231–46. doi: 10.1007/s11103-019-00855-5 (PMC6542783; doi:10.1007/s11103-019-00855-5)

Supplementary Materials

Journal: Plant Molecular Biology

Title: Glutelin subtype-dependent protein localization in rice grain evidenced by immunodetection analyses

Authors: Kei Takahashi^1*^, Hiromi Kohno^1^, Tomomichi Kanabayashi^2^, and Masaki Okuda^1^

Author affiliations:

1, National Research Institute of Brewing, 3-7-1 Kagamiyama, Higashi-hiroshima, Hiroshima, 739-0046, Japan

2, Biopathology Institute Co., Ltd, 1200-2, Ohara Kunisakicho, Kunisaki-city, Oita, 873-0511, Japan

*Corresponding author. Tel.: +81-82-420-8227; Fax: +81-82-420-8228. E-mail address: [k.takahashi@nrib.go.jp](mailto:k.takahashi@nrib.go.jp)


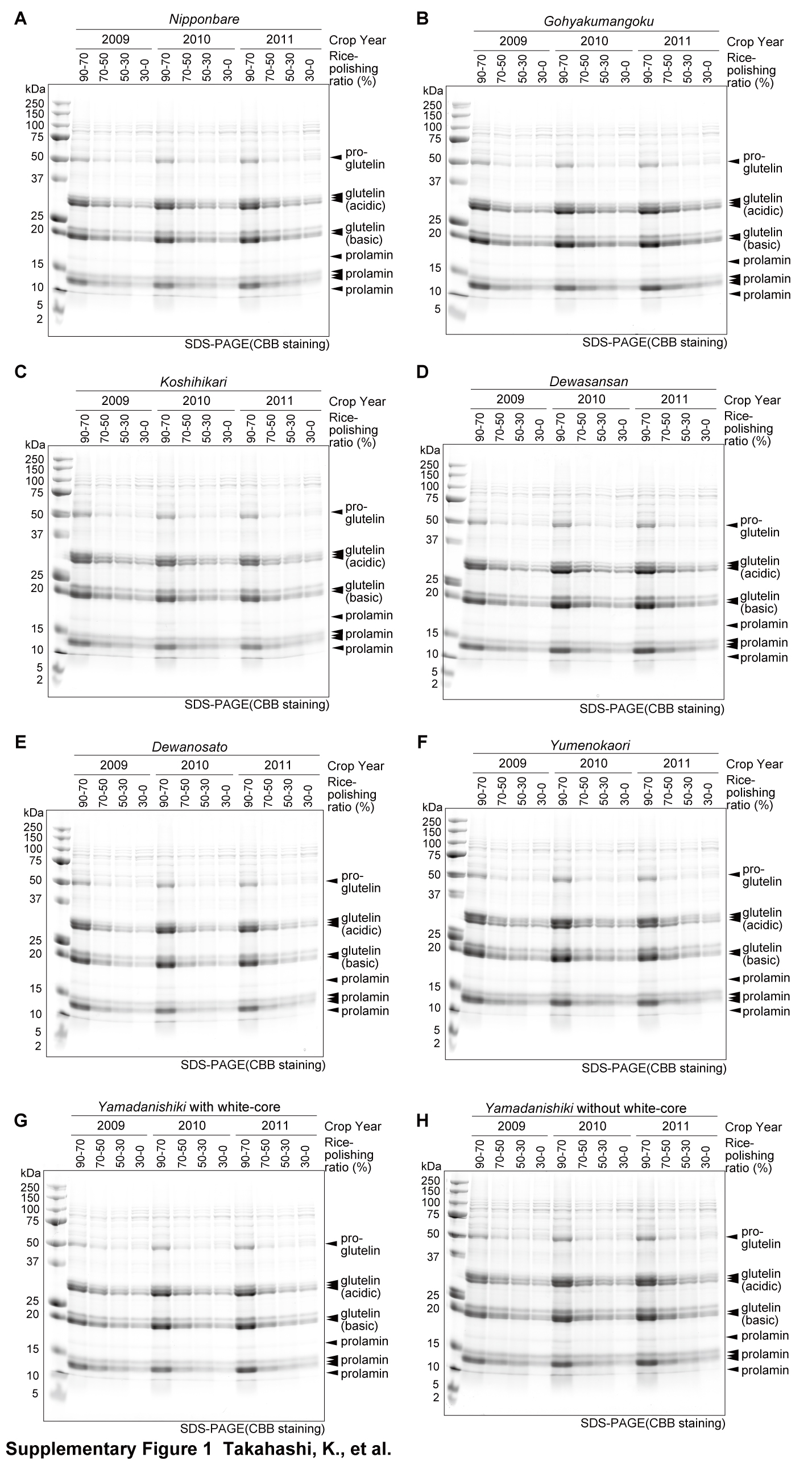


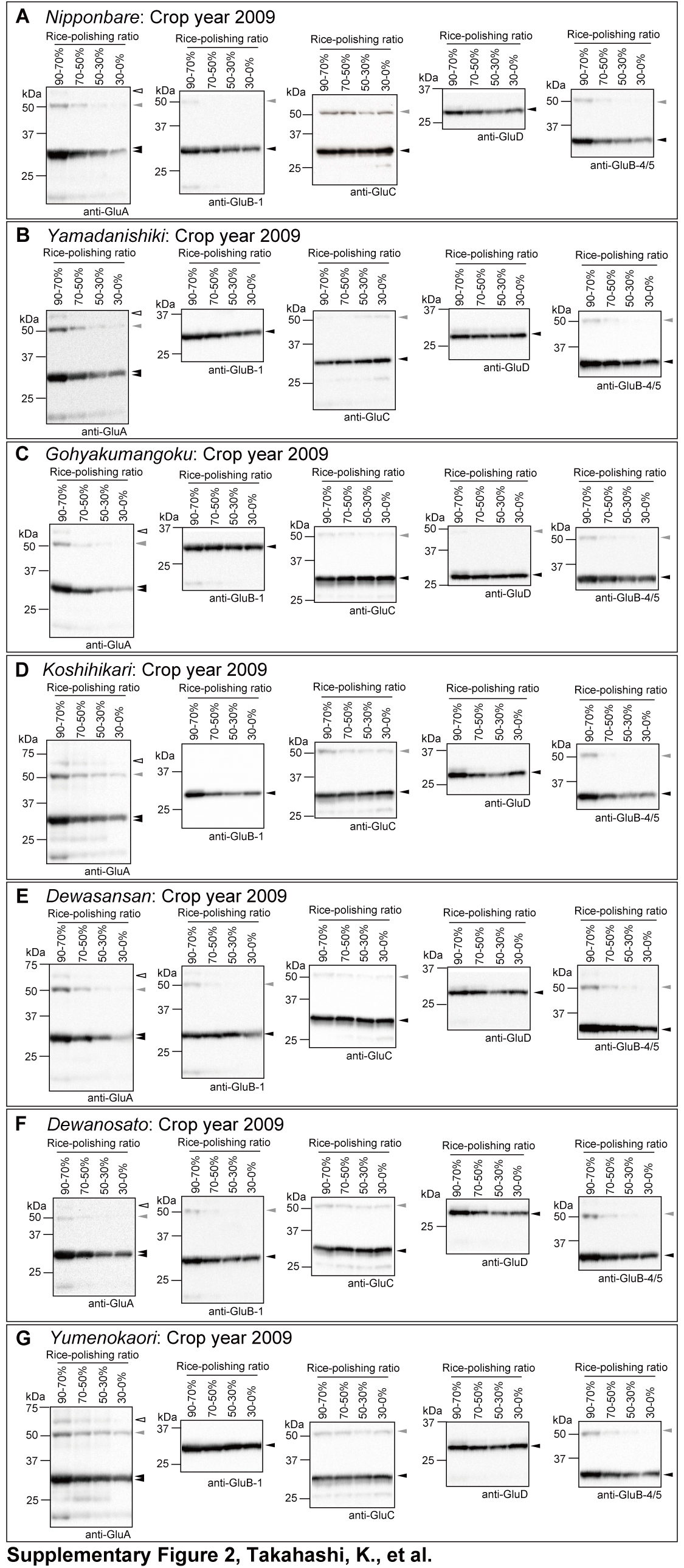


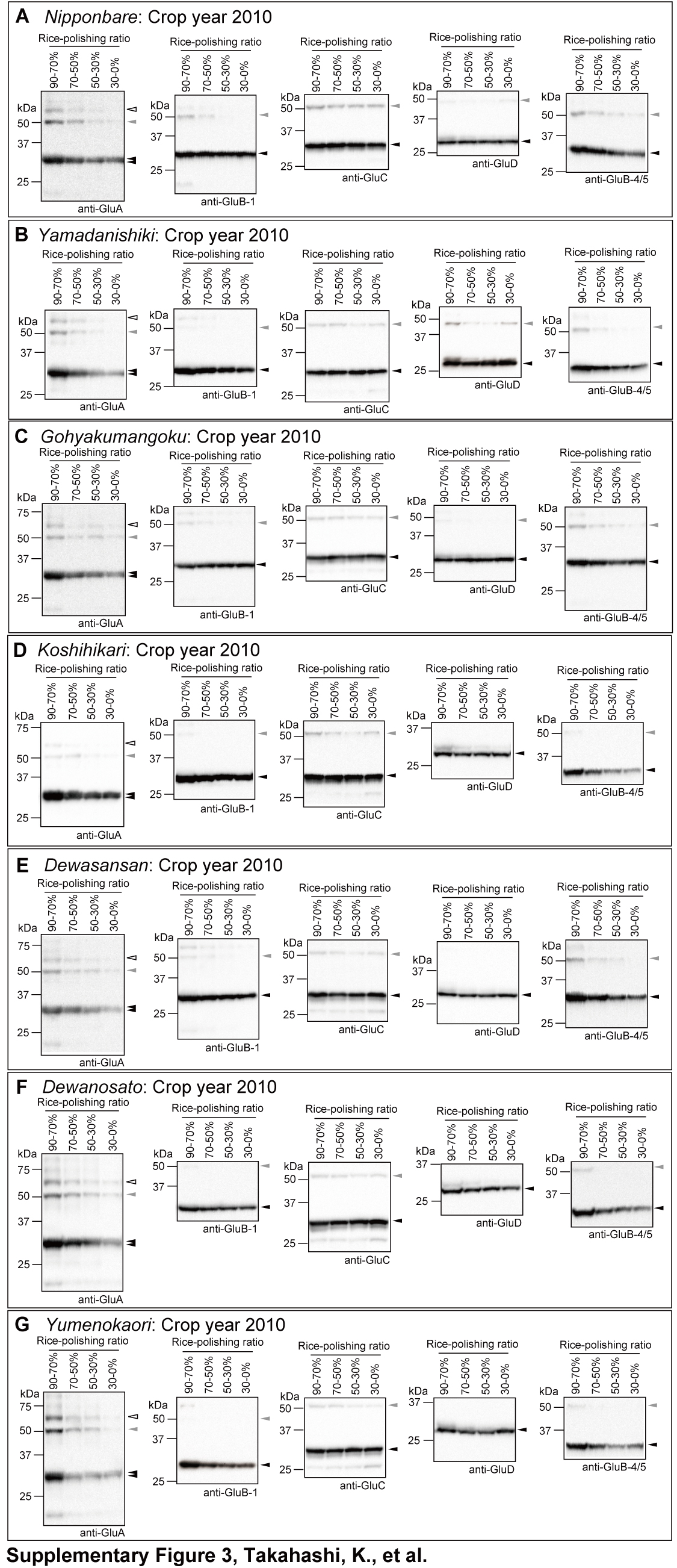


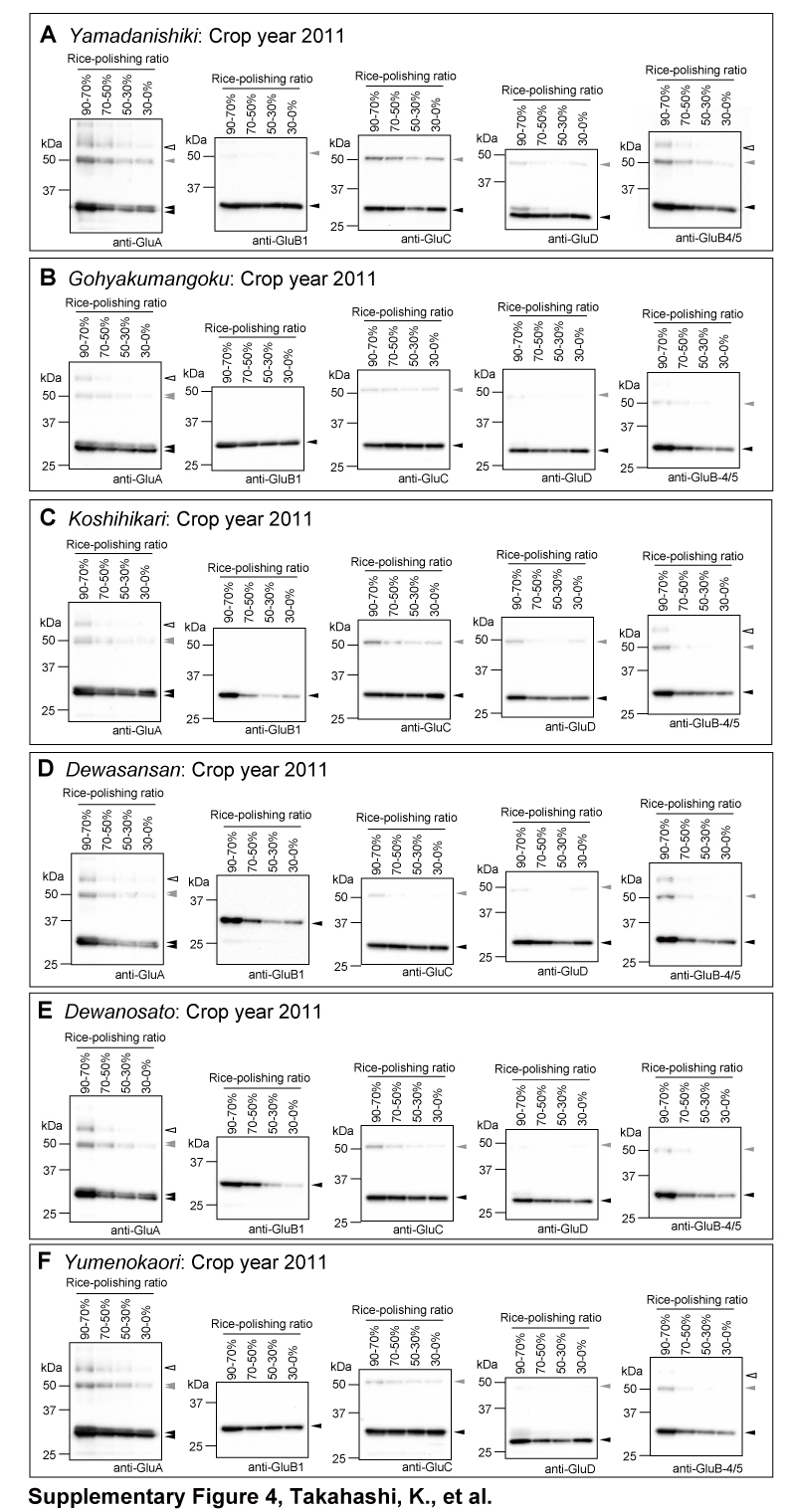


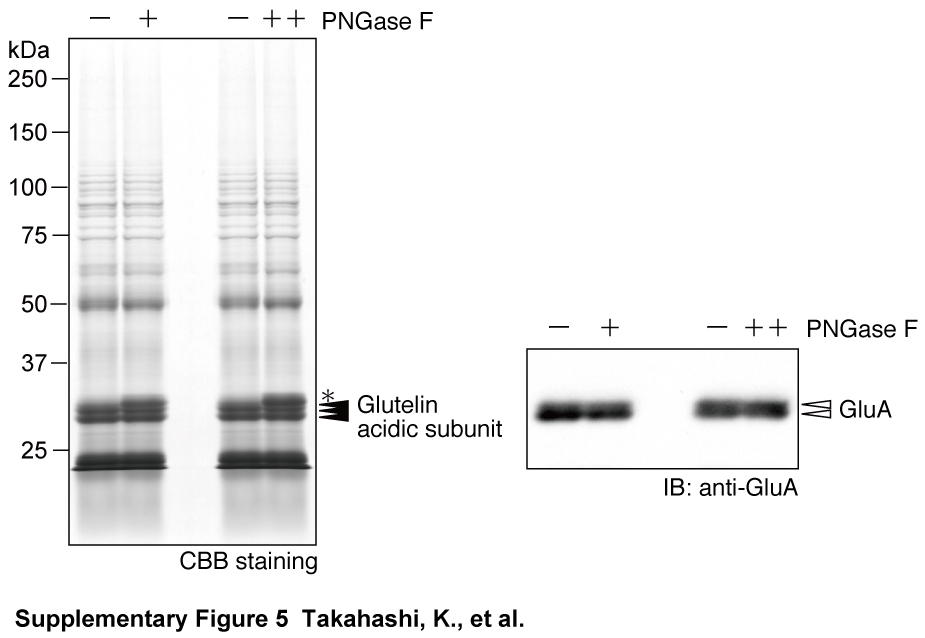


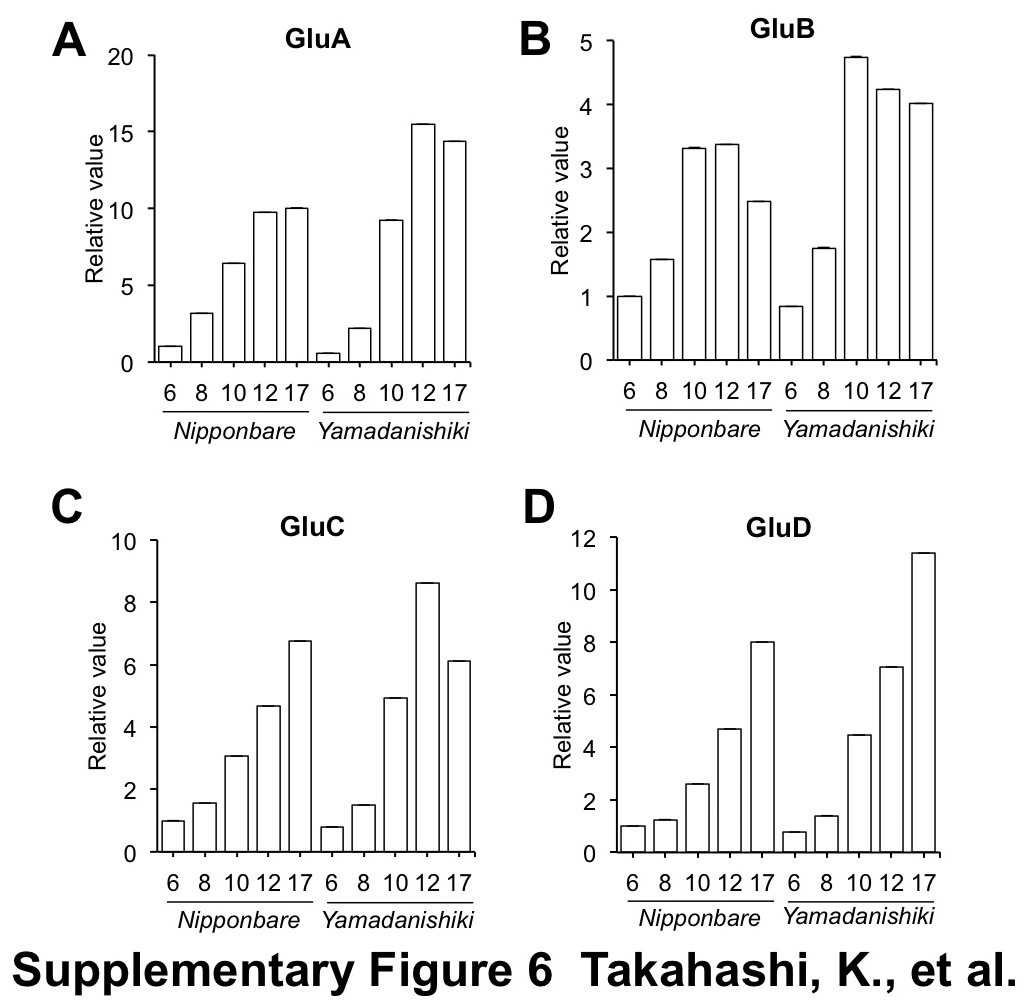


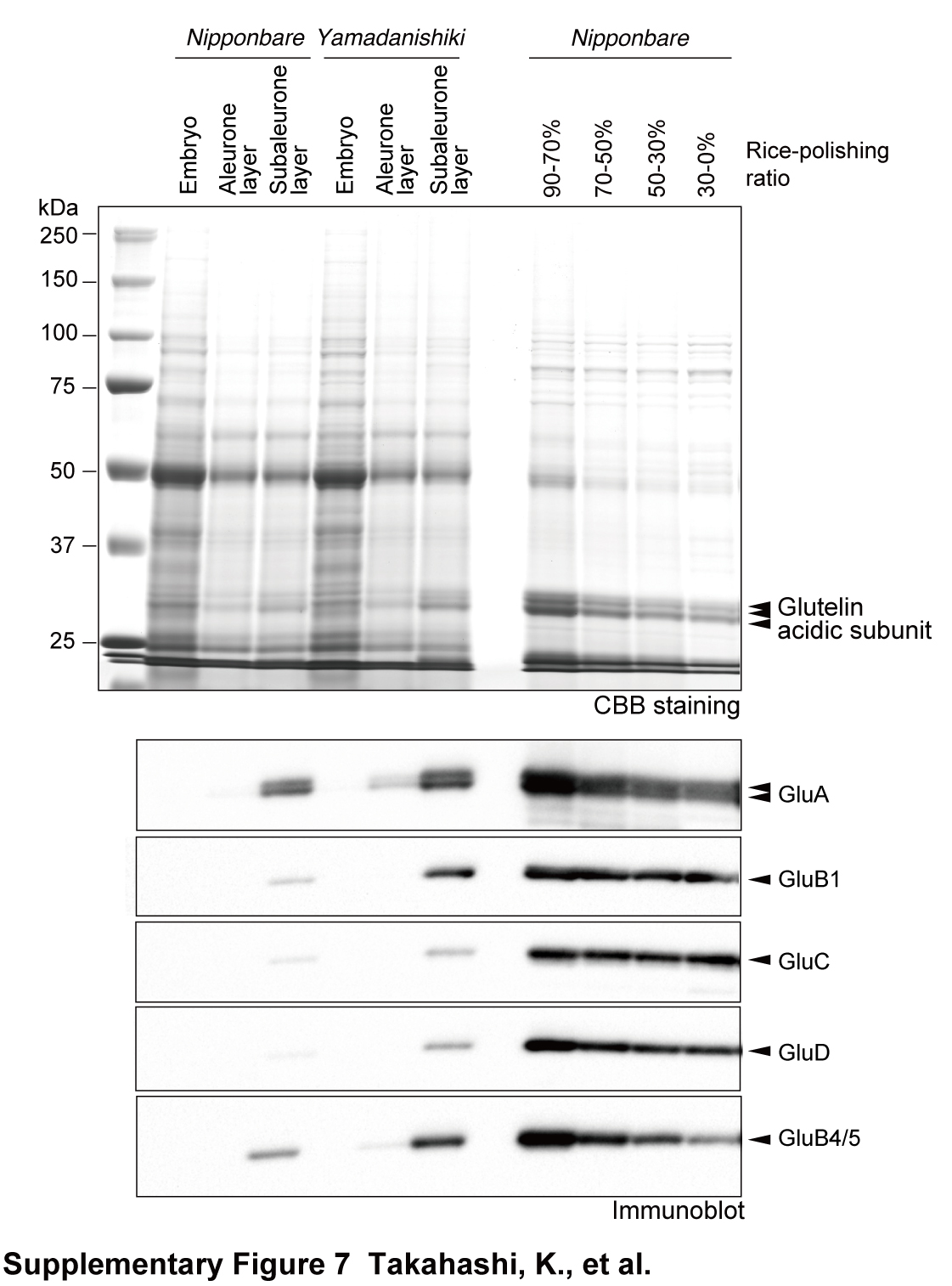

Supplement: Supplementary file 2 — Supplementary material 2 (DOCX 4243 KB) [file 11103_2019_855_MOESM2_ESM.docx]
