# Supplementary material for: Glutelin subtype-dependent protein localization in rice grain evidenced by immunodetection analyses
Source: Plant Mol Biol. 2019 Mar 25;100(3):231–46. doi: 10.1007/s11103-019-00855-5 (PMC6542783; doi:10.1007/s11103-019-00855-5)
Supplement: Supplementary file 3 — Supplementary material 3 (DOCX 14 KB) [file 11103_2019_855_MOESM3_ESM.docx]

**Supplementary Fig. 1. CBB staining of total rice proteins.**

Proteins extracted from fractionated rice powder across three different crop years (2009, 2010, and 2011) were separated by SDS-PAGE and stained with CBB. **a**: *Nipponbare*, **b**: *Gohyakumangoku*, **c**: *Koshihikari*, **d**: *Dewasansan*, **e**: *Dewanosato*, **f**: *Yumenokaori*, **g**: *Yamadanishiki* with white-core, **h**: *Yamadanishiki* without white-core. Each lane contains the same weight of rice powder. Representative data are shown.

**Supplementary Fig. 2. Immunoblot analysis of the glutelin subfamilies of various rice cultivars harvested in 2009.**

Proteins extracted from fractionated rice powder of various rice cultivars were separated by SDS-PAGE and analyzed by immunoblot using anti-GluA, anti-GluB-1, anti-GluC, anti-GluD, and anti-GluB-4/5 antibodies. **a**: *Nipponbare*, **b**: *Yamadanishiki*, **c**: *Gohyakumangoku*, **d**: *Koshihikari*, **e**: *Dewasansan*, **f**: *Dewanosato*, **g**: *Yumenokaori*. Each lane contains the same weight of rice powder. Black triangles indicate glutelin acidic subunit. Gray triangles indicate immature pro-glutelin. Open triangles indicate putative dimeric form of glutelin acidic subunit.

**Supplementary Fig. 3. Immunoblot analysis of the glutelin subfamilies of various rice cultivars harvested in 2010.**

Proteins extracted from fractionated rice powder of various rice cultivars were separated by SDS-PAGE and analyzed by immunoblot using anti-GluA, anti-GluB-1, anti-GluC, anti-GluD, and anti-GluB-4/5 antibodies. **a**: *Nipponbare*, **b**: *Yamadanishiki*, **c**: *Gohyakumangoku*, **d**: *Koshihikari*, **e**: *Dewasansan*, **f**: *Dewanosato*, **g**: *Yumenokaori*. Each lane contains the same weight of rice powder. Black triangles indicate glutelin acidic subunit. Gray triangles indicate immature pro-glutelin. Open triangles indicate putative dimeric form of glutelin acidic subunit.

**Supplementary Fig. 4. Immunoblot analysis of the glutelin subfamilies of various rice cultivars harvested in 2011.**

Proteins extracted from fractionated rice powder of various rice cultivars were separated by SDS-PAGE and analyzed by immunoblot using anti-GluA, anti-GluB-1, anti-GluC, anti-GluD, and anti-GluB-4/5 antibodies. **a**: *Yamadanishiki*, **b**: *Gohyakumangoku*, **c**: *Koshihikari*, **d**: *Dewasansan*, **e**: *Dewanosato*, **f**: *Yumenokaori*. Each lane contains the same weight of rice powder. Black triangles indicate glutelin acidic subunit. Gray triangles indicate immature pro-glutelin. Open triangles indicate putative dimeric form of glutelin acidic subunit.

**Supplementary Fig. 5. PNGase F treatment of rice protein does not affect GluA protein mobility on SDS-PAGE.**

Protein extracted from the 90%-70% fraction of *Yamadanishiki* rice powder was separated by SDS-PAGE. *Left panel*; CBB staining. The plus symbol (+) indicates PNGase F (200 U/lane; New England Biolabs Japan, Tokyo, Japan) treatment. The double plus symbol (++) indicates PNGase F (400 U/lane) treatment. The asterisk at 34 kDa shows PNGase F. *Right panel*; Immunoblot analysis for GluA.

**Supplementary Fig. 6. Temporal expression profiles of glutelin mRNAs in developing rice grains.**

Quantitative real-time PCR analysis of the expression of glutelin subfamilies (GluA, GluB, GluC, and GluD) during developing stages (6, 8, 10, 12, 17 DAF) in *Nipponbare* and *Yamadanishiki* rice seeds. **a**: GluA. **b**: GluB. **c**: GluC. **d**: GluD. Relative value shows the mRNA expression level against the 17S rRNA level as control RNA, and mRNA intensity against *Nipponbare* rice grain at 6 DAF is fixed to 1. Experiments were performed using 8–12 biological replicates. Error bars show three technical replicates.

**Supplementary Fig. 7. Expression level of glutelins in the subaleurone fraction and the starchy endosperm fractions in mature rice grains.**

Proteins extracted and separated by SDS-PAGE from the aleurone layer fraction, the subaleurone layer fraction, the embryo, and 4 starchy endosperm fractions (the rice polishing-ratio of 90–70%, 70–50%, 50–30%, and 30–0%). *Upper panel*; CBB staining. *Lower panel*; Immunoblot analysis for each glutelin subtype. Each lane contains the same weight of tissue.
